# How We Evaluate Postgraduate Medical E-Learning: Systematic Review

**DOI:** 10.2196/13128

**Published:** 2019-04-05

**Authors:** Robert de Leeuw, Anneloes de Soet, Sabine van der Horst, Kieran Walsh, Michiel Westerman, Fedde Scheele

**Affiliations:** 1 Athena Institute for Trans-Disciplinary Research VU University Amsterdam Amsterdam Netherlands; 2 Amsterdam University Medical Center Department of Obstetrics and Gynaecology Vrije Universiteit Amsterdam Netherlands; 3 BMJ Learning London United Kingdom; 4 Department of Internal Medicine Franciscus Gasthuis en Vlietland Hospital Rotterdam Netherlands

**Keywords:** distance education, learning, professional education

## Abstract

**Background:**

Electronic learning (e-learning) in postgraduate medical education has seen a rapid evolution; however, we tend to evaluate it only on its primary outcome or learning aim, whereas its effectiveness also depends on its instructional design. We believe it is important to have an overview of all the methods currently used to evaluate e-learning design so that the preferred method may be identified and the next steps needed to continue to evaluate postgraduate medical e-learning may be outlined.

**Objective:**

This study aimed to identify and compare the outcomes and methods used to evaluate postgraduate medical e-learning.

**Methods:**

We performed a systematic literature review using the Web of Science, PubMed, Education Resources Information Center, and Cumulative Index of Nursing and Allied Health Literature databases. Studies that used postgraduates as participants and evaluated any form of e-learning were included. Studies without any evaluation outcome (eg, just a description of e-learning) were excluded.

**Results:**

The initial search identified 5973 articles, of which we used 418 for our analysis. The types of studies were trials, prospective cohorts, case reports, and reviews. The primary outcomes of the included studies were knowledge, skills, and attitude. A total of 12 instruments were used to evaluate a specific primary outcome, such as laparoscopic skills or stress related to training. The secondary outcomes mainly evaluated satisfaction, motivation, efficiency, and usefulness. We found 13 e-learning design methods across 19 studies (4% 19/418). The methods evaluated usability, motivational characteristics, and the use of learning styles or were based on instructional design theories, such as Gagne’s instructional design, the Heidelberg inventory, Kern’s curriculum development steps, and a scale based on the cognitive load theory. Finally, 2 instruments attempted to evaluate several aspects of a design, based on the experience of creating e-learning.

**Conclusions:**

Evaluating the effect of e-learning design is complicated. Given the diversity of e-learning methods, there are many ways to carry out such an evaluation, and probably, many ways to do so correctly. However, the current literature shows us that we have yet to reach any form of consensus about which indicators to evaluate. There is a great need for an evaluation tool that is properly constructed, validated, and tested. This could be a more homogeneous way to compare the effects of e-learning and for the authors of e-learning to continue to improve their product.

## Introduction

### Background

Electronic learning (e-learning) in postgraduate medical education has seen a rapid evolution [1,2]. Moreover, e-learning has become a central part of education, whether stand-alone, part of hybrid learning, or an essential element in the successful flipped classroom concept [3-5].

Although postgraduate medical e-learning (PGMeL) is becoming part of mainstream education, its effectiveness has been subject to debate. A Cochrane review from 2018 concludes that comparing e-learning with traditional learning seems to reveal little to no difference [6]. Yet, other studies show great benefits with regard to primary outcomes [7,8] or secondary aspects such as environmental impact [9].

A possible reason for this discrepancy can be the heterogeneity in instructional design and other elements of e-learning that are poorly evaluated [10]. PGMeL is frequently evaluated by means of a pre- and posttest of the primary learning aim (eg, new knowledge) [11]. However, every educational instrument has functionalities and elements that are used to optimize its effect. The elements required for a specific e-learning model are defined in the so-called *instructional* *design*. These elements are also called *affordances* and have the purpose of maximizing the effect, effectiveness, and usefulness of an educational instrument [12]. Therefore, the affordance of an instrument is an action made possible by the availability of that tool (eg, interactive videos) [13]. Although several reviews of the effects of e-learning have been carried out, little has been written about the ways in which an e-learning’s instructional design may be evaluated [6,14]. A valuable introduction to the design of e-learning was given by Ellaway and Masters, who provide certain guidelines but no method of evaluation [15]. We believe it is of great importance to have a better insight into the current PGMeL evaluation methods and which outcomes (primary or secondary) are used. The aim of this study was to provide an overview of the outcomes used to evaluate PGMeL and the evaluation methods of the models used. To do so, we first need to provide a working definition of e-learning for this review.

### Electronic Learning Definitions

The definition of e-learning changed with the evolution of the internet, and most definitions fail to describe the subtleties and certain important aspects of e-learning. It does not simply consist of placing documents in an electronic format via the internet. It should encourage interaction, collaboration, and communication, often asynchronously [15]. For this literature review, we have chosen the following, slightly adapted, definition from the study by Sangra et al [16]:

E-learning is an approach to teaching and learning, representing all or part of the educational model applied, that is based on the use of electronic media and devices as tools for improving access to training, communication and interaction and that facilitates the adoption of new knowledge, skills and/or behaviour/attitude.

## Methods

### Study Design

A systematic review was carried out to determine how PGMeL can be evaluated and which outcomes are used. Some studies compared e-learning with other learning methods in trials or cohorts, whereas others were conducted from case reports by authors who evaluated a newly used e-learning method alone. We followed all the steps laid out in the Preferred Reporting Items for Systematic Reviews and Meta-Analyses (PRISMA) guidelines because the risk of bias is not relevant in answering our question [17]; given that we are not looking at the results of the outcomes but, rather, at the content of the outcomes themselves, we did not evaluate the risk of bias.

### Types of Studies and Participants

The types of studies included are trials, reviews, and other descriptive evaluation studies as well as all the studies that evaluated any form of e-learning, as defined above, that have postgraduate medical professionals as a target audience.

### Study Eligibility

The inclusion criteria were as follows:

1. Any e-learning evaluation study (studies without any evaluation outcome were excluded)
2. Postgraduate target audience for the e-learning
3. Published in English
4. Published after the introduction of Web 1.0 (1994)

### Type of Intervention and Outcomes

The type of intervention was any form of e-learning, as discussed in the introduction. Given that the purpose of this review was to overview the kinds of outcomes used, all outcomes were included. We differentiated between primary and secondary outcomes. A primary outcome was defined if the study described the outcome as a primary outcome, if a sample size was calculated based on that outcome, or when the authors defined the outcome in the research question. If it was not clear what the primary outcome was, all outcomes were used as primary outcomes.

### Study Identification

The literature search was performed in November 2017, searching PubMed, Education Resources Information Center, Cumulative Index of Nursing and Allied Health Literature and Web of Science databases separately. The search string was quite extensive and used a combination of Medical Subject Headings terms and a combination of title and abstract keywords. The complete string may be found in Multimedia Appendix 1.

### Study Selection

Working independently and in duplicate, reviewers (RDL, ADS, and SVH) screened all article titles and abstracts. Potentially eligible abstracts and abstracts with disagreement or insufficient information were screened in full text. Disagreements were handled by discussing the full text and the majority counts. The dataset supporting the conclusions of this study is available in the Postgraduate ME Model repository [18].

## Results

### Search Results

**Figure 1:**
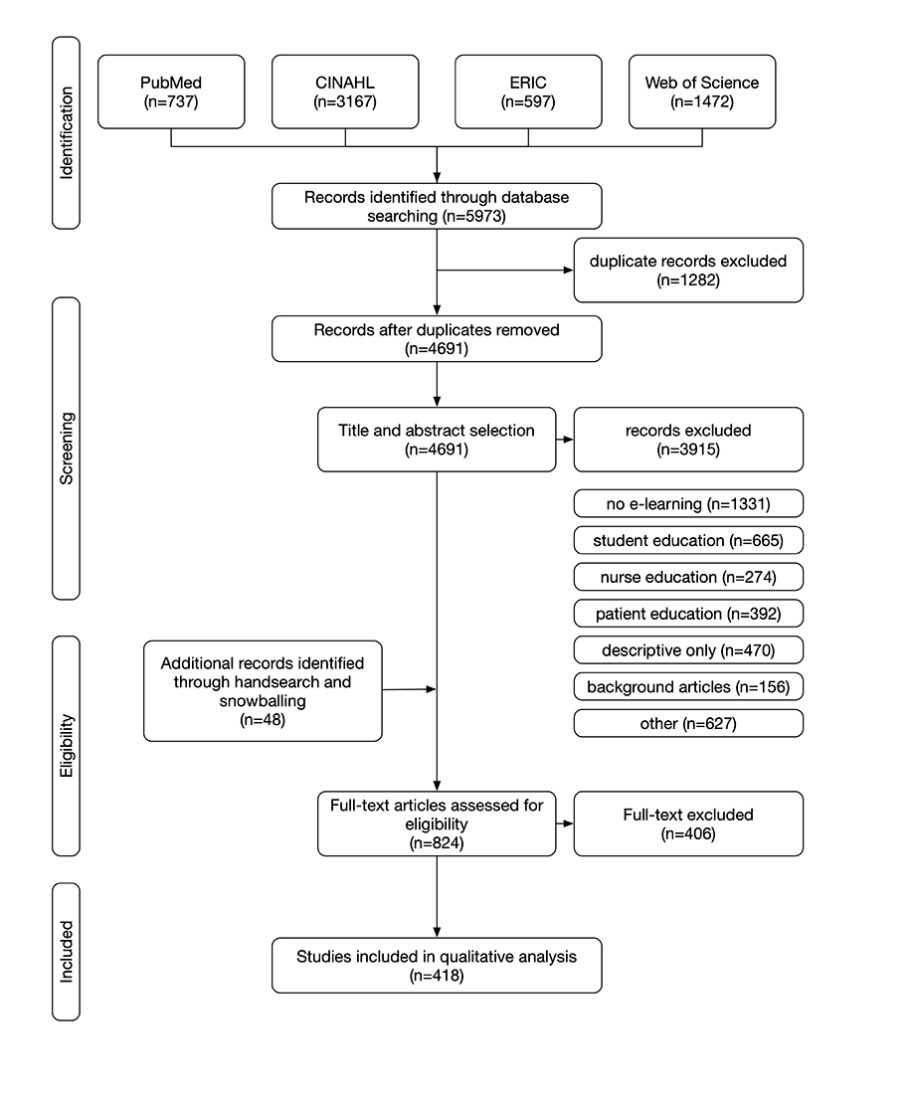
Search and article selection process. e-learning: electronic learning; CINAHL: Cumulative Index of Nursing and Allied Health Literature; ERIC: Education Resources Information Center.

The initial search identified 5973 articles, of which 4691 were left after removing all duplicates. The titles and abstracts were read to determine the relevance, outcomes, and target audience. After handsearching and snowballing, 824 possible studies remained for review. After reading the full texts of these articles, we rejected 406 as not being targeted at the right audience or not evaluating the e-learning but only describing it. We used 418 final articles for our analysis, as shown in the flow diagram in Figure 1, which all evaluated an educational intervention that satisfied our definition of e-learning. For a list of all 418 studies, please refer to Multimedia Appendix 2.

### General Characteristics

**Figure 2:**
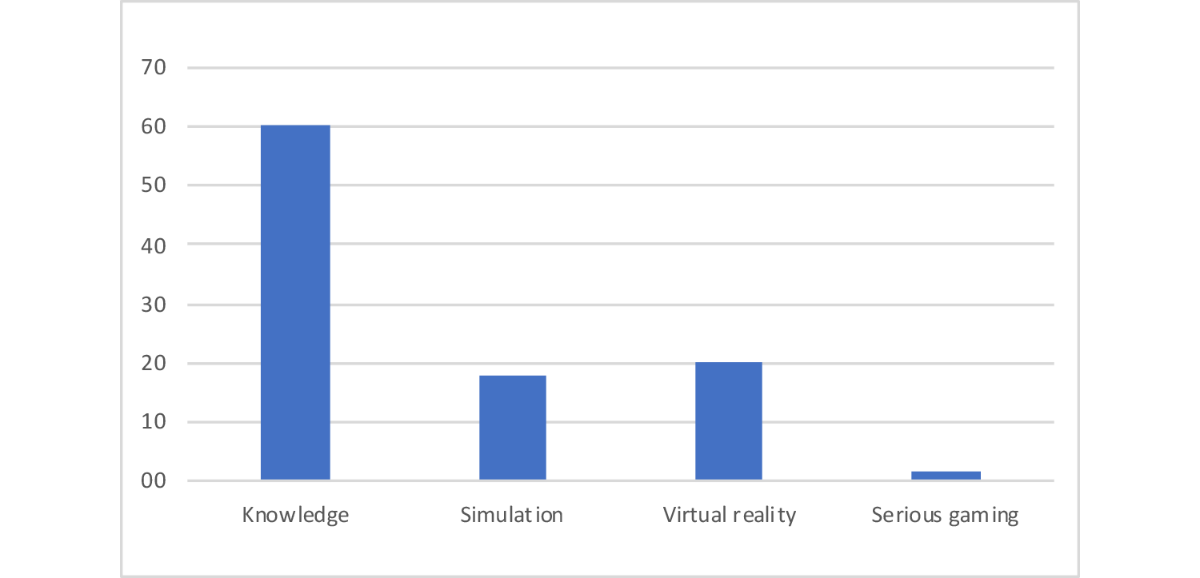
Types of electronic learning (%). Knowledge refers to any acquaintance with facts, truths, or principles. Simulation refers to any form of digital imitation of enactment that is not virtual reality. Virtual reality refers to a simulation of a 3-dimensional environment, experienced or controlled by movement of the body. Serious gaming refers to a learning environment with gamification elements aimed at learning rather than entertainment. e-learning: electronic learning.

**Figure 3:**
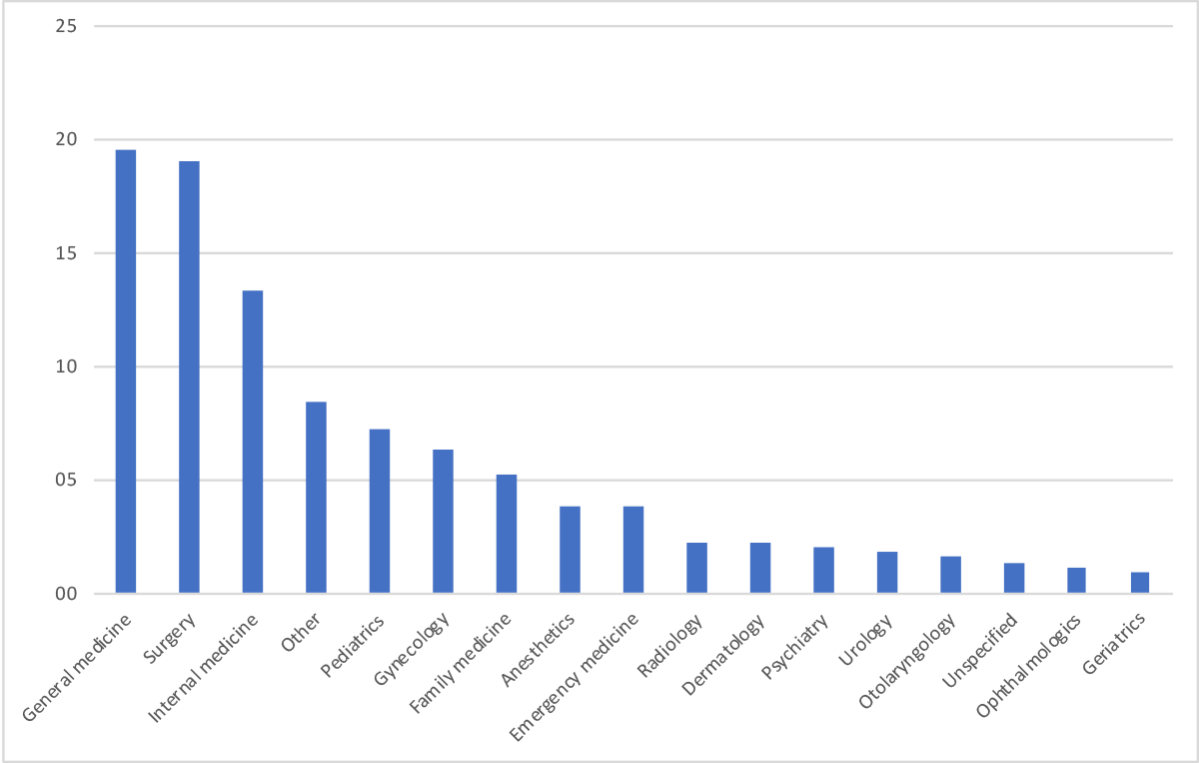
Electronic learning by medical subject (%).

The types of studies were trials (n=201), prospective cohorts (n=110), case reports (n=98), and reviews (n=9). We found a variation of e-learning methods and combined these into 4 categories: serious gaming (n=8), virtual reality (n=90), simulation (n=79), and theoretical knowledge–aimed e-learning (n=241). We added augmented reality into the virtual reality group (Figure 2). Most of the e-learning was created for general medicine (n=86), followed by surgery (n=84), internal medicine (n=59), pediatrics (n=32), gynecology (n=28), and family medicine (n=23; Figure 3). Studies were grouped under general medicine when they were multidisciplinary. A group of 16 studies had no specified target audience. Family medicine was grouped together with primary care.

### Study Outcomes

The learning aims of the included studies were knowledge (n=286), skills (n=130), and attitude (n=2), which reflected the primary outcomes. Knowledge was tested by pre- and postcourse tests, and 12 instruments were used to evaluate an e-learning-specific primary outcome (see Table 1), such as laparoscopic skills or stress related to training.

The secondary outcomes of the studies were both more diverse and more focused on the design (see Table 2). The most prevalent evaluated outcomes were satisfaction (n=99), self-efficacy (n=60), adherence in practice (n=33), and time spent (n=32). Overall, 28 studies had some sort of qualitative evaluation, such as focus discussions or personal interviews. To prevent too diverse a series of outcomes, we grouped comparable outcomes together. Therefore, satisfaction can be measured by using a Likert scale but also by asking if someone would recommend the e-learning to other residents. Adherence in practice can be self-reported practice change or objective changes in practice, for example, subscription practice. We used the term *self-efficacy* for each form of self-assessed confidence, understanding, or comfort in clinical or theoretical settings.

A total of 5 studies used Kirkpatrick’s levels of evaluation. These levels were more used as secondary outcomes of the learning aim than as a design evaluation method [29,33-36]. Kirkpatrick described a 4-level framework of evaluation for measuring the effectiveness of training or human performance technology programs originally aimed at corporate human resources [37]. The levels are reaction, learning, behavior, and results. Aitken et al evaluated their radiology e-learning material based on the first 2 levels, using the framework to build an evaluation questionnaire [34]. Sim et al focused on learning, behavior change, and impact on workplace by quantitative pre-, mid- and postmodule surveys; qualitative Wseb-based discussions; and short facilitator meetings [33]. In 2016, Bowe et al evaluated their e-learning program by means of the Kirkpatrick framework, but a narrative review provided them with the 3 other evaluation tools discussed below as well [29]. Finally, Patel et al undertook a review to establish the effectiveness of simulation in interventional radiology and evaluated which level of Kirkpatrick’s hierarchy the studies reached, with only 1 reaching level 4. No proper validation of PGMeL has been carried out, and there are many concerns about the overgeneralization and misunderstandings that compromise its evaluation [38]. One study by Sears et al [39] used Robert and McDonald’s revision of Kirkpatrick’s levels, where the third and fourth levels fall into an overall *practice* domain and a new level, *value*, is added to better suit current technologies and continuing education approaches.

### Electronic Learning Design Evaluation Methods and Theories

Overall, 19 studies (4%) used some form of tool to evaluate the e-learning design, and 13 tools were described in these studies. These 19 studies alone provided us with the methods and theories at which our initial research question was aimed.

Two instruments focused on usability, namely, the System Usability Scale (SUS) and the Software Usability Measurement Inventory (SUMI).

### The System Usability Scale (n=5)

This is a 10-item questionnaire developed by Brooke that measures the usability of computer systems in 3 domains: effectiveness, efficiency, and satisfaction. It has been freely available since 1986 and has been cited in more than 1200 publications [40]. Davids et al used the SUS first to evaluate an e-learning resource for electrolyte and acid-base disorders [41] and again in 2014 to evaluate the effect of improving usability [42]. The SUS was also used by Gorrindo et al [43], Diehl et al [44], and Gillespie in 2017 [45].

**Table 1:** Discipline of skill-specific outcome measurement tools.

Name	Evaluation topic	Reference
Vandenberg and Kuse mental rotations test	Laparoscopic skills	Ahlborg [19]
Arthroscopic Surgery Skill Evaluation Tool	Arthroscopic skills	Waterman [20]
Stanford Microsurgery and Resident Training Scale	Microsurgery skills	Satterwhite [21]
Global Operative Assessment of Laparoscopic Skills	Laparoscopic skills	Rinewalt [22]
McGill Inanimate System for Training and Evaluation of Laparoscopic Skills	Laparoscopic skills	Martinez [23]
Objective Structured Assessment of Technical Skills	Laparoscopic skills	Tomaz [24]
Evaluating the attitude toward research tests	Attitude toward testing	Pelayo [25]
Survey of Attitudes toward Achieving Competency in Practice-Based Learning and Improvement and System-Based Practice	Managed care competencies and attitude	Yedidia [26]
Attitude, belief, and Behavior survey regarding domestic violence	Attitude to domestic violence	Harris [27]
State-Trait Anxiety Inventory	Stress	Samakar [28]
Mini-Mental State Exam	Stress	Tomaz [24]
Attitude Toward Health Care Teams Scale	Teamwork	Bowe [29] Leipzig [30]
Assessment of Care for the Vulnerable Elderly	Elderly care	Holmboe [31]
Cumulative sum analysis for colorectal histology	Histology	Patel [32]

**Table 2:** Secondary outcomes.

Outcome	Statistics, n (%)
Satisfaction	88 (19.9)
Self-efficacy	60 (13.6)
Adherence in practice	31 (7.0)
Long-term follow-up	28 (6.3)
Qualitative evaluation	28 (6.3)
Time spent	27 (6.1)
Skills	25 (5.7)
Attitude	20 (4.5)
Usefulness	16 (3.6)
Efficiency	8 (1.8)
Confidence	8 (1.8)
Usability	8 (1.8)
Acceptability	6 (1.4)
Preference	6 (1.4)
Costs	5 (1.1)
Presentation quality	5 (1.1)
Knowledge	4 (0.9)
Motivation	4 (0.9)
Stress	3 (0.7)
Patient satisfaction	2 (0.5)
Agreement	1 (0.2)
Discomfort	1 (0.2)
Overall reaction	1 (0.2)
Participation	1 (0.2)
Readiness to change	1 (0.2)
Screening percentage	1 (0.2)
Cognitive load	1 (0.2)

### The Software Usability Measurement Inventory (n=1)

According to Deraniyagala et al, there are multiple approaches to measuring usability, but the gold standard is the SUMI because of its extensive validations and long track record of success in evaluation [46]. It consists of a 50-item questionnaire devised in accordance with psychometric practice and was inspired by the 1993 ISO 9241 definition by Kiralowski and Corbett [47].

A total of 3 instruments attempted to evaluate the motivational characteristics of the design.

### The Motivated Strategies for Learning Questionnaire (n=1)

Ahlborg et al used a few items from the Motivated Strategies for Learning Questionnaire to evaluate self-efficacy [19] and Cook et al validated the entire questionnaire [48]. It consists of a self-reported, Likert scale instrument developed by Pintrich et al in 1993, which aims to assess the motivation and use of learning strategies by college students [49]. Cook et al concluded that the scores are reliable and offer meaningful outcomes for residents in a Web-based course.

### Keller’s Instructional Attention, Relevance, Confidence, and Satisfaction Motivation Model (n=2)

This proposes to assess the motivational characteristics of instructional materials or courses using an Attention, Relevance, Confidence, and Satisfaction (ARCS) model of motivation and was validated by Cook et al with 124 internal medicine residents [50]. Although the data were limited, they support the validity of the survey. Kawamura et al used the system as well to determine factors of motivation in serious gaming [51].

### Instructional Materials Motivation Survey (n=1)

Cooke et al validated the Instructional Materials Motivation Survey (IMMS) to assess the motivational characteristics of a course [50]. The IMMS is an instrument developed by Keller using his ARCS model. The aim of the tool is to improve a course design generally or to adapt a course to an individual’s needs.

The 2 scales focused on the use of learning styles as described in the following sections.

### The Learning Style Index (n=2)

The Learning Style Index [52,53], developed in 1988 by Richard Felder and Linda Silverman, is designed to capture the most important learning style of engineering students, differentiated by 4 dimensions (active-reflective, visual-verbal, sensing-intuitive, and sequential-global) [54]. Cook et al evaluated whether the preferred learning style had any effect on a Web-based course and questions. Cognitive and learning styles had no apparent influence on learning outcomes [53].

### Riding’s Cognitive Style Analysis (n=1)

Riding’s Cognitive Style Analysis (RCSA) determines whether an individual has a particular cognitive style or a preferred way of processing information [53]. The RCSA test measures the cognitive style on a verbal-imagery dimension and a holistic-analytic dimension [55].

A total of 4 tools were based on previous instructional design theories: Gagne’s instructional design, the Heidelberg inventory, Kern’s curriculum development steps, and a scale based on cognitive load theory.

### Gagne’s Events Instructions (n=1)

The instructional design by Gagne et al has been a classic in learning since 1974 and is a general, instructional design theory [56]. It has 9 parts, mirroring Gagne’s idea of the cognitive stages associated with adult learning [57]. The model is used as a framework for designing any adult education instrument.

### Heidelberg Inventory for the Evaluation of Teaching (n=1)

The Heidelberg Inventory for the Evaluation of Teaching [58] is a. standardized, psychometric questionnaire for the didactic quality assessment of the whole program. It consists of 13 domains and 42 items/questions and was developed to evaluate teaching methods for German undergraduate students [59].

### Kern’s 6-Step Curriculum Development for Medical Education (n=1)

This approach [60], described by Kern et al in 2009, aimed to create a planned educational experience with a logical, systematic approach [61].

### Learner’s Scale (n=1)

This series of scales [62] is composed of learner satisfaction, self-efficacy, mental effort, and time on task. The questions used for these scales are based on cognitive load principles and multimedia learning, which are based on the work by Clark and Mayer [63] and van Merrienboer [64].

Finally, 2 instruments attempted to evaluate several aspects of a design, based on the experience of creating e-learning.

### The 10 Golden Rules for Software Design (n=2)

Created to help in designing software in medical education, this [36,65] starts with a 51-item questionnaire based on the Context, Input, Process, and Product model by Stufflebeam [66]; the Convenience, Relevance, Individualization, Self-assessment, Interest, Speculation, and Systematic criteria [67]; and Kirkpatrick’s 4 levels of evaluation. The questionnaire was then piloted and used to evaluate an interactive distance education course in obstetrics and gynecology [36]. From the qualitative data, 10 common items were identified and represented in the form of *10 golden rules*.

### Quality Improvement Knowledge Application Tool-Revised (n=1)

A revision of the original Quality Improvement Knowledge Application Tool, validated to assess practice-based learning and the system-based practice of residents, the Quality Improvement Knowledge Application Tool-Revised (QIKATR) [29,68] consists of 3 subjects—aim, measure, and change—and participants are asked to score the presented scenarios on these subjects.

Apart from these evaluation methods, we found 4 studies that did not evaluate e-learning but did use evaluation methods to create their e-learning. These used instruments to create e-learning with a focus on outcomes, motivation, and technology acceptance:

### The Formative Process and Outcome Evaluation Tool by Dunet

Dunet et al [69] described the evaluation process by which they created a course—formative evaluation (content and design), process evaluation (knowledge gain, motivation, and usefulness), and outcome evaluation.

### The Website Motivational Analysis Checklist

The authors reviewed an education database and did not find any validated tools. Therefore, they used the Website Motivational Analysis Checklist [70], which was originally created to assess service-based commercial websites in 2000 [71].

### Davis’s Technology Acceptance Model and Laurillard’s Model of Interactive Dialogue

A realistic review by Wong et al [72] identified these 2 main theories as having a significant focus on perceived advantage, ease of use, interactivity, and feedback.

Finally, Rosen et al describe a statistical tool to apply to the study of teleoperation, human manipulation actions, and manufacturing applications (*Hidden Markov Model*), which they suggest might also be useful for other evaluation methods [73].

The abovementioned evaluation models all evaluate certain domains, a summary of which is presented in Tables 3 and 4 as an overview. In the final column, we have added the domains evaluated by de Leeuw et al in previous studies [74].

**Table 3:** Domains and methods for evaluating postgraduate medical electronic learning design (part 1).

Factor	Riding’s Cognitive Style Analysis	Kern’s six steps	Motivated Strategies For Learning Questionnaire	Software Usability Measurement Inventory	Dunet model	Website Motivational Analysis Checklist	Davis's model	de Leeuw quality indicators
Learning aims/objectives	—^a^	x^b^	x	—	x	x	x	x
Measurement of performance	—	x	x	—	x	—	—	x
Aim for change/transfer to the job	—	x	—	—	x	—	—	x
Satisfaction	—	—	—	x	x	—	—	x
Usability and control	—	—	—	x	x	x	x	x
Integration or recall of prior learning	—	x	x	—	x	—	—	x
Confidence	—	—	—	—	x	—	—	x
Suitability/usefulness/relevance/helpfulness	—	x	x	x	x	x	x	x
Attention	—	—	x	—	—	—	—	x
Sensing or intuitive learning	x	—	—	—	—	—	—	—
Visual or verbal learning	x	—	—	—	—	—	—	—
Active or reflective learning	x	—	—	—	—	—	—	—
Sequential or global learning	x	—	—	—	—	—	—	—
Content accountability	—	—	—	—	x	x	—	x
Multimedia use	—	x	—	—	x	x	—	x
Problem-based setting	—	—	x	—	—	—	—	x
Impetus for use/motivation	—	x	x	x	x	x	—	x
Costs	—	—	—	—	—	—	—	x
Feedback and interactivity	—	x	x	—	—	x	x	x
Challenge	—		x	—	—	x	—	x
Commitment and maintenance	—	x	x	—	—	—	—	x
Implementation	—	x	—	—	x	—	—	x
Rehearsal	—	—	x	—	—	—	—	x
Time management	—	—	x	—	x	—	—	x
Tasks	—	—	x	—	—	—	—	x
Efficiency	—	—	—	x	—	—	—	x
User expectation	—	—	—	—	—	—	x	—

^a^Factor present in the model.

^b^Factor not present in the model.

**Table 4:** Domains and methods for evaluating postgraduate medical electronic learning design (part 2).

Factor	Quality Improvement Knowledge Application Tool-Revised (model of improvement)	System Usability Scale	Instructional Materials Motivation Survey	Attention, Relevance, Confidence, and Satisfaction motivation model	Index of learning styles	10 golden rules	Gagne’s events instructions	Heidelberg inventory for the evaluation of teaching
Learning aims/objectives	x^a^	—^b^	—	—	—	x	x	x
Measurement of performance	x	—	—	—	—	—	x	x
Aim for change/transfer to the job	x	x	—	—	—	—	x	x
Satisfaction	—	x	x	x	—	x	—	x
Usability and control	—	x	—	—	—	x	—	x
Integration or recall of prior learning	—	x	—	—	—	—	x	x
Confidence	—	x	x	x	—	—	—	—
Suitability/usefulness/relevance/helpfulness	—	x	x	x	—	x	x	x
Attention	—	—	—	x	—	x	x	—
Sensing or intuitive learning	—	—	—	—	x	—	x	—
Visual or verbal learning	—	—	—	—	x	x	x	—
Active or reflective learning	—	—	—	—	x	—	x	—
Sequential or global learning	—	—	—	—	x	—	x	—
Content accountability	—	—	—	—	—	x	x	x
Multimedia use	—	—	—	—	—	x	x	x
Problem-based setting	—	—	—	—	—	x	x	—
Impetus for use/motivation	—	—	—	—	—	x	—	x
Costs	—	—	—	—	—	x	x	—
Feedback and interactivity	—	—	—	—	—	—	x	x
Challenge	—	—	—	—	—	—	—	x
Commitment and maintenance	—	—	—	—	—	—	—	x
Implementation	—	—	—	—	—	—	—	—
Rehearsal	—	—	—	—	—	—	—	—
Time management	—	—	—	—	—	—	—	—
Tasks	—	—	—	—	—	—	—	—
Efficiency	—	—	—	—	—	—	—	—
User expectation	—	—	—	—	—	—	—	—

^a^Factor present in the model.

^b^Factor not present in the model.

## Discussion

### Principal Findings

There are many ways to evaluate PGMeL, and evaluation is clearly focused on the outcomes of the intervention. We found 14 e-learning-specific and 3 general primary outcomes, 27 secondary outcomes, and 13 evaluations tools. More than half of the studies (60%) had knowledge gain as their primary aim, which is almost the same finding as that in the 2016 review by Taveira-Gomes et al [2], who looked at all kinds of education. We are looking at PGMeL only and found that 38% were simulation and virtual reality studies. This kind of e-learning was not mentioned specifically in the study by Taveira-Gomes et al but might be comparable with the skills outcome (14.6%). The difference could be the result of postgraduates’ need to undertake more task- and real-life-related e-learning, as described in our focus groups [74]. The experts from that study emphasized real-world translation as an important factor for PGMeL. Looking at the outcomes of the studies, Seagull identified similar domains in surgical simulation studies [75]. Self-efficacy, satisfaction, relevance/adherence in practice, and attitude are frequently used as outcomes of e-learning in both our study and that by Seagull et al. Table 1 shows a list of methods used to evaluate an outcome, which may be laparoscopic skills, attitude, or stress. They focus on the defined outcome rather than the method used to achieve it. Many other instruments are available (such as the critical thinking index [76]), but they are either not yet used in a PGMeL e-learning evaluation setting or were not revealed by our search.

Our research question asked which evaluation methods are used. As mentioned above, only 4% used a method, and of those methods, we can differentiate between theories and instruments.

Of the theories, Kirkpatrick’s hierarchy is the most used to evaluate or create e-learning. A 2017 review by Patel et al evaluated the effectiveness of simulation in interventional radiology training [35]. It also found different studies using the levels of Kirkpatrick’s hierarchy to establish or evaluate the success of the e-learning. Of the educational instructional theories, 2 are leading in e-learning in general and were also found in our studies: Gagne’s principles of instructional design and Mayers and Clark’s e-learning and the science of instruction, also referred to as *Mayers’ multimedia learning*. Mayers and Clark base their instructions on the cognitive load theory, which provides design guidelines based on a model of human cognitive architecture. Cook et al validated a cognitive load index in 2017 [77]. The last theory from our search is from Kern’s curriculum development for medical education: a 6-step approach. All these theories are either based on education in general (eg, the work of Gagne and Mayer) or medical education (eg, the work of Kirkpatrick and Kern), but none of the theories are aimed at PGMeL. They are used to evaluate PGMeL but not specifically aimed at this audience. The Heidelberg inventory for the evaluation of teaching is even aimed at undergraduate students and only used because of the lack of a better alternative [59].

Apart from these theories, some instruments focused on 1 aspect of the design. Although these instruments have a specific focus, Table 1 shows that they cover a wider range of domains. Instruments that aim to evaluate the course as a whole are QIKATR, 10 golden rules, and Dunet’s formative process and outcome evaluation tool. The QIKATR is an answer to the Accreditation Council for Graduate Medical Education, which required practice-based learning and improvement. It is a description of 3 scenarios depicting quality problems. Although the domains are not very specific (describe the aim, measure the effect, and require change), they are aimed at postgraduates and provide a good basis for any education. Conversely, they are not aimed at e-learning education [68]. In 2002, Jha et al created an e-learning model for gynecology called the Distance Interactive Learning in Obstetrics and Gynaecology. They then evaluated the e-learning, and the lessons learned were described as 10 golden rules [65]. These golden rules are aimed at postgraduates and are specific to e-learning. The most significant downside of these rules is that they are based on 1 e-learning experience only; therefore, they may be incomplete or biased by the single case that created the fundament. Finally, Dunet’s formative process and outcome evaluation tool is the result of an evaluation plan based on the experience of creating a hemochromatosis training course for continuing education credits and continuing nurse education. The course has been intensively evaluated by several experts, and the key findings can be summarized in 2 domains: instructional design and content, and usability and navigation. Although aimed at postgraduate education and specific to e-learning, it is based on 1 course only and might, therefore, lack important domains and items that were not available in that course.

As demonstrated in Tables 3 and 4, Gagne’s science of instruction covers most of the domains. Our search did not identify any e-learning evaluation methods that are not expert opinion–based or the result of a single evaluation and aimed at PGMeL. A previous study by our group identified all these domains in literature [78], then evaluated their relevance with the focus groups [74] and an international Delphi [79]. The domains are added in the last column of Tables 3 and 4, which illustrates that all domains, except learning styles, are identified as important in these studies. The learning styles were identified in the review, but the effect of learning style–specific education is disappointing [53]. The conclusion was that it was better not to evaluate the learning style but to offer a diversity in each e-learning [74].

### Strengths and Limitations

We believe that the biggest limitation is our search. Had we included papers not aimed at postgraduate education, we would have found many more papers and evaluation models. We could also have included papers that did not actually evaluate a course but only described a theoretical model. However, our research question asked not what is available but what is actually used. We also believed in differentiating between graduate and postgraduate education, hence the choice in our search. However, we also believe that making this distinction is a strength. This paper provides an insight into the diversity of evaluating e-learning and how little is known of and targeted at the right audience. Almost all quality models signify the importance of knowing your target audience but our evaluation tools do not.

### Conclusions

It may be asked what comes next. We have reached the point at which we should stop evaluating only the outcomes of e-learning as an educational intervention and start evaluating the e-learning design that goes with it. However, to do so, we need a validated instrument to help us assess the nuances of all the different electronic education instruments. We believe that our previous studies have provided us with validated content for such a tool [74,79] and that this paper emphasizes the need for such a system.

PGMeL is evaluated in very diverse ways, almost exclusively based on its outcomes or learning aims. Although there is a need to evaluate the e-learning itself as well, we lack the instruments to do so. This paper provides an overview of available instruments; however, they are not aimed at postgraduate medical education, not expert opinion–based, or the result of lessons learned from a single case study. With the increasing ease of creating and distributing e-learning, the need for a content-validated evaluation tool is of ever greater importance.

## Appendix

Multimedia Appendix 1 Search string in detail.

Multimedia Appendix 2 Search results (sorted by year).

## Abbreviations

ARCS: Attention, Relevance, Confidence, and Satisfaction
e-learning: electronic learning
IMMS: Instructional Materials Motivation Survey
PGMeL: postgraduate medical e-learning
QIKATR: Quality Improvement Knowledge Application Tool-Revised
SUMI: Software Usability Measurement Inventory
SUS: System Usability Scale
