# Supplementary material for: How We Evaluate Postgraduate Medical E-Learning: Systematic Review
Source: JMIR Med Educ. 2019 Apr 5;5(1):e13128. doi: 10.2196/13128 (PMC6473211; doi:10.2196/13128)
Supplement: Multimedia Appendix 1 [file mededu_v5i1e13128_app1.pdf]

Appendix A – search string

Databases used

- PubMed
- CINAHL
- ERIC
- Web of Science

1. postgrad med

("Education, Medical, Graduate"[Mesh] OR "Internship and Residency"[Mesh] OR (house[tiab] AND officer*[tiab]) OR resident*[tiab] OR residency[tiab] OR intern[tiab] OR interns[tiab] OR internship*[tiab] OR trainee*[tiab] OR registrar*[tiab] OR postgradua*[tiab] OR post gradua*[tiab] OR cme[tiab] OR cmes[tiab] OR continuous medical education*[tiab])

2. e-learning tools

"Computer-Assisted Instruction"[Mesh] OR "Education, Distance"[Mesh] OR e-learning[Title/Abstract] OR electronic learning[Title/Abstract] OR distance education[Title/Abstract] OR technology-enhanced learning[Title/Abstract] OR tele-learning[Title/Abstract] OR distance learning[Title/Abstract] OR (("Computer Communication Networks"[Mesh] OR "User-Computer Interface"[Mesh] OR "Multimedia"[Mesh] OR "Cell phones"[Mesh] OR "Computers, handheld"[Mesh] OR "Mobile Applications"[Mesh] OR "Internet"[Mesh] OR app[tiab] OR apps[tiab] OR smartphone*[tiab] OR phone application*[tiab] OR telephone application*[tiab] OR mobile application*[tiab] OR mobile technolog*[tiab] OR internet*[tiab] OR world wide web*[tiab] OR webbased[tiab] OR web based[tiab] OR webportal*[tiab] OR web portal*[tiab] OR ipad[tiab] OR ipads[tiab] OR sms[tiab] OR mms[tiab] OR text messag*[tiab] OR ussd[tiab] OR pda[tiab] OR laptop*[tiab] OR palmtop*[tiab] OR palm top*[tiab] OR personal digital assistant*[tiab] OR multimedia[tiab] OR iphone*[tiab] OR android[tiab] OR game*[tiab] OR gaming[tiab] OR gamification[tiab] OR whatsapp*[tiab] OR wearable*[tiab] OR social media[tiab] OR "online social network"[tiab] OR "online social networks"[tiab] OR facebook[tiab] OR serious gam*[tiab] OR electronic*[tiab] OR computer based[tiab] OR online[tiab] OR virtual*[tiab]) AND ("Learning"[Mesh] OR "Education, Professional"[Mesh] OR learning[tiab] OR education*[tiab] OR instructi*[tiab] OR simulat*[tiab]))
"Computer-Assisted Instruction"[Majr] OR "Education, Distance"[Majr] OR e-learning[Ti] OR electronic learning[Ti] OR distance education[Ti] OR technology-enhanced learning[Ti] OR tele-learning[Ti] OR distance learning[Ti] OR (("Computer Communication Networks"[Majr] OR "User-Computer Interface"[Majr] OR "Multimedia"[Majr] OR "Cell phones"[Majr] OR "Computers, handheld"[Majr] OR "Mobile Applications"[Majr] OR "Internet"[Majr] OR app[ti] OR apps[ti] OR smartphone*[ti] OR phone application*[ti] OR telephone application*[ti] OR mobile application*[ti] OR mobile technolog*[ti] OR internet*[ti] OR world wide web*[ti] OR

webbased[ti] OR web based[ti] OR webportal*[ti] OR web portal*[ti] OR ipad[ti] OR ipads[ti] OR sms[ti] OR mms[ti] OR text messag*[ti] OR ussd[ti] OR pda[ti] OR laptop*[ti] OR palmtop*[ti] OR palm top*[ti] OR personal digital assistant*[ti] OR multimedia[ti] OR iphone*[ti] OR android[ti] OR game*[ti] OR gaming[ti] OR gamification[ti] OR whatsapp*[ti] OR wearable*[ti] OR social media[ti] OR "online social network"[ti] OR "online social networks"[ti] OR facebook[ti] OR serious gam*[ti] OR electronic*[ti] OR computer based[ti] OR online[ti] OR virtual*[ti]) AND ("Learning"[Majr] OR "Education, Professional"[Majr] OR learning[ti] OR education*[ti] OR instructi*[ti] OR simulat*[ti]))

3. **RCT's, SR's**

((random*[tiab] AND (controlled[tiab] OR control[tiab] OR placebo[tiab] OR versus[tiab] OR vs[tiab] OR group[tiab] OR groups[tiab] OR comparison[tiab] OR compared[tiab] OR arm[tiab] OR arms[tiab] OR crossover[tiab] OR cross-over[tiab]) AND (trial[tiab] OR study[tiab])) OR ((single[tiab] OR double[tiab] OR triple[tiab]) AND (masked[tiab] OR blind*[tiab])))

OR

((review*[tiab] OR search*[tiab] OR survey*[tiab] OR handsearch*[tiab] OR hand-search*[tiab]) AND (databa*[tiab] OR data-ba*[tiab] OR bibliograph*[tiab] OR electronic*[tiab] OR medline*[tiab] OR pubmed*[tiab] OR embase*[tiab] OR Cochrane[tiab] OR cinahl[tiab] OR psycinfo[tiab] OR psychinfo[tiab] OR cinhal[tiab] OR "web of science"[tiab] OR "web of knowledge"[tiab] OR ebsco[tiab] OR ovid[tiab] OR mrct[tiab] OR metaregist*[tiab] OR meta-regist*[tiab] OR ((predetermined[tiab] OR pre-determined[tiab]) AND criteri*[tiab]) OR apprais*[tiab] OR inclusion criteri*[tiab] OR exclusion criteri*[tiab])) OR (review[pt] AND systemat*[tiab]) OR "systematic review"[tiab] OR "systematic literature"[tiab] OR "integrative review"[tiab] OR "integrative literature"[tiab] OR "evidence-based review"[tiab] OR "evidence-based overview"[tiab] OR "evidence-based literature"[tiab] OR "evidence-based survey"[tiab] OR "literature search"[tiab] OR ((systemat*[ti] OR evidence-based[ti]) AND (review*[ti] OR literature[ti] OR overview[ti] OR survey[ti])) OR "data synthesis"[tiab] OR "evidence synthesis"[tiab] OR "data extraction"[tiab] OR "data source"[tiab] OR "data sources"[tiab] OR "study selection"[tiab] OR "methodological quality"[tiab] OR "methodologic quality"[tiab] OR cochrane database syst rev[ta] OR meta-analy*[tiab] OR metaanaly*[tiab] OR metanaly*[tiab] OR meta-analysis[pt] OR meta-synthesis[tiab] OR metasynthesis[tiab] OR meta-study[tiab] OR metastudy[tiab] OR metaethnograph*[tiab] OR meta-ethnograph*[tiab] OR Technology Assessment, Biomedical[mh] OR hta[tiab] OR health technol assess [ta] OR evid rep technol assess summ[ta] OR health technology assessment[tiab]) OR ((review*[ot] OR search*[ot] OR survey*[ot] OR handsearch*[ot] OR hand-search*[ot]) AND (databa*[ot] OR data-ba*[ot] OR bibliograph*[ot] OR electronic*[ot] OR medline*[ot] OR pubmed*[ot] OR embase*[ot] OR cochrane[ot] OR cinahl[ot] OR psycinfo[ot] OR psychinfo[ot] OR cinhal[ot] OR "web of science"[ot] OR "web of knowledge"[ot] OR ebsco[ot] OR ovid[ot] OR mrct[ot] OR metaregist*[ot] OR meta-regist*[ot] OR ((predetermined[ot] OR pre-determined[ot]) AND criteri*[ot]) OR apprais*[ot] OR inclusion criteri*[ot] OR exclusion criteri*[ot]) OR (review[pt] AND systemat*[ot]) OR "systematic review"[ot] OR "systematic literature"[ot] OR "integrative review"[ot] OR "integrative literature"[ot] OR "evidence-based review"[ot] OR "evidence-based overview"[ot] OR "evidence-based literature"[ot] OR "evidence-based survey"[ot] OR

"literature search"[ot] OR ((systemat*[ti] OR evidence-based[ti]) AND (review*[ti] OR literature[ti] OR overview[ti] OR survey[ti])) OR "data synthesis"[ot] OR "evidence synthesis"[ot] OR "data extraction"[ot] OR "data source"[ot] OR "data sources"[ot] OR "study selection"[ot] OR "methodological quality"[ot] OR "methodologic quality"[ot] OR meta-analy*[ot] OR metaanaly*[ot] OR metanaly*[ot] OR meta-analysis[pt] OR meta-synthesis[ot] OR metasynthesis[ot] OR meta-study[ot] OR metastudy[ot] OR metaethnograph*[ot] OR meta-ethnograph*[ot] OR hta[ot] OR health technology assessment[ot])
