# Supplementary material for: How We Evaluate Postgraduate Medical E-Learning: Systematic Review
Source: JMIR Med Educ. 2019 Apr 5;5(1):e13128. doi: 10.2196/13128 (PMC6473211; doi:10.2196/13128)
Supplement: Multimedia Appendix 2 [file mededu_v5i1e13128_app2.pdf]

Appendix B – search results (sorted by year)

1. Chew, F.S. and J.G. Smirniotopoulos, Teaching skeletal radiology with use of computer-assisted instruction with interactive videodisc. J Bone Joint Surg Am, 1995. 77(7): p. 1080-6.
2. Lee, W., et al., Interactive multimedia for prenatal ultrasound training. Obstet Gynecol, 1995. 85(1): p. 135-40.
3. Ohrn, M.A., J.H. van Oostrom, and W.L. van Meurs, A comparison of traditional textbook and interactive computer learning of neuromuscular block. Anesth Analg, 1997. 84(3): p. 657-61.
4. Devitt, P., et al., Evaluation of a computer based package on electrocardiography. Aust N Z J Med, 1998. 28(4): p. 432-5.
5. Lewis, Y.L., et al., Changes in residents' attitudes and achievement after distance learning via two-way interactive video. Fam Med, 1998. 30(7): p. 497-500.
6. Peugnet, F., P. Dubois, and J.F. Rouland, Virtual reality versus conventional training in retinal photocoagulation: a first clinical assessment. Comput Aided Surg, 1998. 3(1): p. 20-6.
7. Tuggy, M.L., Virtual reality flexible sigmoidoscopy simulator training: impact on resident performance. J Am Board Fam Pract, 1998. 11(6): p. 426-33.
8. O'Toole, R.V., et al., Measuring and developing suturing technique with a virtual reality surgical simulator. J Am Coll Surg, 1999. 189(1): p. 114-27.
9. Schwid, H.A., et al., Use of a computerized advanced cardiac life support simulator improves retention of advanced cardiac life support guidelines better than a textbook review. Crit Care Med, 1999. 27(4): p. 821-4.
10. Bell, D.S., et al., Self-study from web-based and printed guideline materials. A randomized, controlled trial among resident physicians. Annals of Internal Medicine, 2000. 132(12): p. 938-946.
11. Curran, V.R., et al., Web-based continuing medical education. (II): Evaluation study of computer-mediated continuing medical education. J Contin Educ Health Prof, 2000. 20(2): p. 106-19.
12. Francis, B., et al., Assessment of online continuing dental education in North Carolina. J Contin Educ Health Prof, 2000. 20(2): p. 76-84.
13. Issenberg, S.B., et al., Bedside cardiology skills training for the physician assistant using simulation technology. Perspective on Physician Assistant Education, 2000. 11(2): p. 99-103.
14. Jordan, J.A., et al., A comparison between randomly alternating imaging, normal laparoscopic imaging, and virtual reality training in laparoscopic psychomotor skill acquisition. Am J Surg, 2000. 180(3): p. 208-11.
15. Kronz, J.D., et al., A web-based tutorial improves practicing pathologists' gleason grading of images of prostate carcinoma specimens obtained by needle biopsy: Validation of a new medical education paradigm. Cancer, 2000. 89(8): p. 1818-1823.
16. Kronz, J.D., et al., Pathology residents' use of a Web-based tutorial to improve Gleason grading of prostate carcinoma on needle biopsies. Hum Pathol, 2000. 31(9): p. 1044-50.
17. Pelayo-Alvarez, M., et al., Feasibility analysis of a personalized training plan for learning research methodology. Med Educ, 2000. 34(2): p. 139-45.
18. Colt, H.G., S.W. Crawford, and O. Galbraith, 3rd, Virtual reality bronchoscopy simulation: a revolution in procedural training. Chest, 2001. 120(4): p. 1333-9.
19. Grantcharov, T.P., et al., Virtual reality computer simulation: An objective method for the evaluation of laparoscopic surgical skills. Surgical Endoscopy, 2001. 15(3): p. 242-244.
20. Jefford, M., K.A. Phillips, and M.H. Tattersall, An online educational facility for medical oncology trainees: www.vmotg.org. J Clin Oncol, 2001. 19(9): p. 2566-9.
21. Marsh, C.M., et al., Design and effectiveness of a computer-based continuing education program for orthodontists. Angle Orthod, 2001. 71(1): p. 71-5.
22. Ramshaw, B.J., et al., The role of multimedia interactive programs in training for laparoscopic procedures. Surg Endosc, 2001. 15(1): p. 21-7.

23. Schwid, H.A., et al., Screen-based anesthesia simulation with debriefing improves performance in a mannequin-based anesthesia simulator. Teach Learn Med, 2001. 13(2): p. 92-6.
24. Cahill, D., et al., Evaluation of an online postgraduate education programme. Med Teach, 2002. 24(4): p. 425-28.
25. Harris Jr, J.M., et al., Can internet-based education improve physician confidence in dealing with domestic violence? Family Medicine, 2002. 34(4): p. 287-292.
26. Hillenbr, K.M. and P.G. Larsen, Effect of an educational intervention about breastfeeding on the knowledge, confidence, and behaviors of pediatric resident physicians. Pediatrics, 2002. 110(5): p. 7p-7p.
27. Jha, V., S. Duffy, and S. McAleer, Evaluation of distance interactive learning in obstetrics and gynaecology (DIALOG). BJOG, 2002. 109(4): p. 456-61.
28. Moller, T., et al., Interactive training for the management of breast cancer in general practice in Europe. J Cancer Educ, 2002. 17(1): p. 19-23.
29. Nizard, J., M. Duyme, and Y. Ville, Teaching ultrasound-guided invasive procedures in fetal medicine: learning curves with and without an electronic guidance system. Ultrasound Obstet Gynecol, 2002. 19(3): p. 274-7.
30. Pedowitz, R.A., J. Esch, and S. Snyder, Evaluation of a virtual reality simulator for arthroscopy skills development. Arthroscopy, 2002. 18(6): p. E29.
31. Rosen, J., et al., Task decomposition of laparoscopic surgery for objective evaluation of surgical residents' learning curve using hidden Markov model. Comput Aided Surg, 2002. 7(1): p. 49-61.
32. Rowe, R. and R.A. Cohen, An evaluation of a virtual reality airway simulator. Anesth Analg, 2002. 95(1): p. 62-6, table of contents.
33. Seymour, N.E., et al., Virtual reality training improves operating room performance: results of a randomized, double-blinded study. Ann Surg, 2002. 236(4): p. 458-63; discussion 463-4.
34. Wiecha, J. and N. Barrie, Collaborative online learning: a new approach to distance CME. Acad Med, 2002. 77(9): p. 928-9.
35. Wilhelm, D.M., et al., Assessment of basic endoscopic performance using a virtual reality simulator. Journal of the American College of Surgeons, 2002. 195(5): p. 675-681.
36. Allen, M., et al., Videoconferencing for practice-based small-group continuing medical education: feasibility, acceptability, effectiveness, and cost. J Contin Educ Health Prof, 2003. 23(1): p. 38-47.
37. Gerson, L.B. and J. Van Dam, A prospective randomized trial comparing a virtual reality simulator to bedside teaching for training in sigmoidoscopy. Endoscopy, 2003. 35(7): p. 569-75.
38. Lemaire, E. and G. Greene, A comparison between three electronic media and in-person learning for continuing education in physical rehabilitation. Journal of telemedicine and telecare, 2003. 9(1): p. 17-22.
39. Mikail, C.N., E. Hearney, and B. Nemesure, Increasing physician awareness of the common uses and contraindications of herbal medicines: utility of a case-based tutorial for residents. J Altern Complement Med, 2003. 9(4): p. 571-6.
40. Moorthy, K., et al., Evaluation of virtual reality bronchoscopy as a learning and assessment tool. Respiration, 2003. 70(2): p. 195-199.
41. Browne, L., et al., Comparing lecture and e-learning as pedagogies for new and experienced professionals in dentistry. British Dental Journal, 2004. 197(2): p. 95-97.
42. Casebeer, L., et al., Standardizing evaluation of on-line continuing medical education: physician knowledge, attitudes, and reflection on practice. J Contin Educ Health Prof, 2004. 24(2): p. 68-75.
43. Chung, S., et al., Efficacy of an educational web site for educating physicians about bioterrorism. Academic Emergency Medicine, 2004. 11(2): p. 143-148.
44. Di Giulio, E., et al., Training with a computer-based simulator achieves basic manual skills required for upper endoscopy: a randomized controlled trial. Gastrointest Endosc, 2004. 60(2): p. 196-200.

45. Grantcharov, T.P., et al., Randomized clinical trial of virtual reality simulation for laparoscopic skills training. Br J Surg, 2004. 91(2): p. 146-50.
46. Johnson, C.E., et al., Learning management systems: technology to measure the medical knowledge competency of the ACGME. Med Educ, 2004. 38(6): p. 599-608.
47. Mahnke, C.B., et al., Comparison of two educational interventions on pediatric resident auscultation skills. Pediatrics, 2004. 113(5): p. 1331-5.
48. Rossi, J.V., et al., Virtual vitreoretinal surgical simulator as a training tool. Retina, 2004. 24(2): p. 231-6.
49. Ahlberg, G., et al., Virtual reality colonoscopy simulation: a compulsory practice for the future colonoscopist? Endoscopy, 2005. 37(12): p. 1198-204.
50. Allison, J.J., et al., Multicomponent Internet continuing medical education to promote chlamydia screening. Am J Prev Med, 2005. 28(3): p. 285-90.
51. Bello, G., et al., Online vs live methods for teaching difficult airway management to anesthesiology residents. Intensive Care Med, 2005. 31(4): p. 547-52.
52. Bridgemohan, C.F., et al., Teaching paediatric residents about learning disorders: use of standardised case discussion versus multimedia computer tutorial. Medical Education, 2005. 39(8): p. 797-806.
53. Cook, A., et al., Prospective evaluation of remote, interactive videoconferencing to enhance urology resident education: the genitourinary teleteaching initiative. J Urol, 2005. 174(5): p. 1958-60.
54. Cook, D.A., et al., Web-based learning in residents' continuity clinics: a randomized, controlled trial. Acad Med, 2005. 80(1): p. 90-7.
55. Curran, V.R. and L. Fleet, A review of evaluation outcomes of web-based continuing medical education. Med Educ, 2005. 39(6): p. 561-7.
56. Felsher, J.J., et al., Validation of a flexible endoscopy simulator. Am J Surg, 2005. 189(4): p. 497-500.
57. Fordis, M., et al., Comparison of the instructional efficacy of Internet-based CME with live interactive CME workshops: a randomized controlled trial. JAMA, 2005. 294(9): p. 1043-51.
58. Gold, J.P., et al., Evaluation of web-based learning tools: lessons learned from the thoracic surgery directors association curriculum project three-year experience. Ann Thorac Surg, 2005. 80(3): p. 802-9; discussion 809-10.
59. Markova, T., L.M. Roth, and J. Monsur, Synchronous distance learning as an effective and feasible method for delivering residency didactics. Fam Med, 2005. 37(8): p. 570-5.
60. Ricci, M.A., et al., The use of telemedicine for delivering continuing medical education in rural communities. Telemed J E Health, 2005. 11(2): p. 124-9.
61. Ro, C.Y., et al., The LapSim: a learning environment for both experts and novices. Stud Health Technol Inform, 2005. 111: p. 414-7.
62. Schijven, M.P., et al., The Eindhoven laparoscopic cholecystectomy training course--improving operating room performance using virtual reality training: results from the first E.A.E.S. accredited virtual reality trainings curriculum. Surg Endosc, 2005. 19(9): p. 1220-6.
63. Scholer, S.J., et al., A multimedia violence prevention program increases pediatric residents' and childcare providers' knowledge about responding to childhood aggression. Clinical Pediatrics, 2005. 44(5): p. 413-417.
64. Wolters, R., et al., Effects of distance learning on clinical management of LUTS in primary care: a randomised trial. Patient Educ Couns, 2005. 59(2): p. 212-8.
65. Wong, R.W. and H. Lochnan, Online simulations of ambulatory care for medical residents. Med Educ, 2005. 39(5): p. 527-8.
66. Anderson, J.M., et al., Simulating extracorporeal membrane oxygenation emergencies to improve human performance. Part I: methodologic and technologic innovations. Simul Healthc, 2006. 1(4): p. 220-7.
67. Casebeer, L., et al., Evaluation of an online bioterrorism continuing medical education course. J Contin Educ Health Prof, 2006. 26(2): p. 137-44.

68. Chen, J.S., et al., Validation of a computer-based bronchoscopy simulator developed in Taiwan. J Formos Med Assoc, 2006. 105(7): p. 569-76.
69. Cohen, J., et al., Multicenter, randomized, controlled trial of virtual-reality simulator training in acquisition of competency in colonoscopy. Gastrointest Endosc, 2006. 64(3): p. 361-8.
70. Cook, D.A., et al., Internet-based continuing medical education...Fordis M, King JE, Ballantyne CM et al. Comparison of the instructional efficacy of Internet-based CME with live interactive CME workshops: a randomized controlled trial. JAMA. 2005;294:1043-1051. JAMA: Journal of the American Medical Association, 2006. 295(7): p. 758-759.
71. Curran, V., et al., Evaluation of learning outcomes in Web-based continuing medical education. Acad Med, 2006. 81(10 Suppl): p. S30-4.
72. Fakih, M.G., et al., A Web-based course on infection control for physicians in training: an educational intervention. Infect Control Hosp Epidemiol, 2006. 27(7): p. 704-8.
73. Finlay, K., et al., A web-based test of residents' skills in diagnostic radiology. Can Assoc Radiol J, 2006. 57(2): p. 106-16.
74. Friedl, R., et al., Development and prospective evaluation of a multimedia teaching course on aortic valve replacement. Thorac Cardiovasc Surg, 2006. 54(1): p. 1-9.
75. Goldmann, K. and T. Steinfeldt, Acquisition of basic fiberoptic intubation skills with a virtual reality airway simulator. J Clin Anesth, 2006. 18(3): p. 173-8.
76. Hackethal, A., M. Immenroth, and T. Burger, Evaluation of target scores and benchmarks for the traversal task scenario of the Minimally Invasive Surgical Trainer-Virtual Reality (MIST-VR) laparoscopy simulator. Surg Endosc, 2006. 20(4): p. 645-50.
77. Hassan, I., et al., Novices in surgery are the target group of a virtual reality training laboratory. Eur Surg Res, 2006. 38(2): p. 109-13.
78. Kale, S. and B. Richardson, The effective use of e-learning in postgraduate health-care students...including commentary by Moule P and Gotthardt M. International Journal of Therapy & Rehabilitation, 2006. 13(7): p. 299-303.
79. Karlinsky, H., et al., Workplace injury management: using new technology to deliver and evaluate physician continuing medical education. Journal of Occupational Rehabilitation, 2006. 16(4): p. 719-730.
80. Larsen, C.R., et al., Objective assessment of gynecologic laparoscopic skills using the LapSimGyn virtual reality simulator. Surg Endosc, 2006. 20(9): p. 1460-6.
81. LoRusso, A.P., M.J. Bassignani, and J.A. Harvey, Enhanced teaching of screening mammography using an electronic format. Acad Radiol, 2006. 13(6): p. 782-8.
82. Matsumoto, E.D., K.T. Pace, and D.A.H. RJ, Virtual reality ureteroscopy simulator as a valid tool for assessing endourological skills. Int J Urol, 2006. 13(7): p. 896-901.
83. Naldi, L., et al., Feasibility of a web-based continuing medical education program in dermatology: the DermoFAD experience in Italy. Dermatology, 2006. 213(1): p. 6-11.
84. Schiefer, U., et al., K-Train--a computer-based, interactive training program with an incorporated certification system for practicing kinetic perimetry: evaluation of acceptance and success rate. Graefes Arch Clin Exp Ophthalmol, 2006. 244(10): p. 1300-9.
85. Short, L.M., Z.J. Surprenant, and J.M. Harris, Jr., A community-based trial of an online intimate partner violence CME program. Am J Prev Med, 2006. 30(2): p. 181-5.
86. Triola, M., et al., A randomized trial of teaching clinical skills using virtual and live standardized patients. J Gen Intern Med, 2006. 21(5): p. 424-9.
87. Aggarwal, R., et al., Proving the effectiveness of virtual reality simulation for training in laparoscopic surgery. Ann Surg, 2007. 246(5): p. 771-9.
88. Ahlberg, G., et al., Proficiency-based virtual reality training significantly reduces the error rate for residents during their first 10 laparoscopic cholecystectomies. Am J Surg, 2007. 193(6): p. 797-804.
89. Ashar, B.H., T.N. Rice, and S.D. Sisson, Physicians' understanding of the regulation of dietary supplements. Arch Intern Med, 2007. 167(9): p. 966-9.

90. Autti, T., et al., E-learning is a well-accepted tool in supplementary training among medical doctors: an experience of obligatory radiation protection training in healthcare. Acta radiologica (Stockholm, Sweden : 1987), 2007. 48(5): p. 508-513.
91. Banks, E., et al., An interactive computer program for teaching residents pap smear classification, screening and management guidelines: a pilot study. J Reprod Med, 2007. 52(11): p. 995-1000.
92. Bell, D.S., J. Higa, and C.M. Mangione, Learning and retention from an online tutorial among resident physicians. AMIA ... Annual Symposium proceedings / AMIA Symposium. AMIA Symposium, 2007: p. 870.
93. Berry, M., et al., Porcine transfer study: virtual reality simulator training compared with porcine training in endovascular novices. Cardiovasc Intervent Radiol, 2007. 30(3): p. 455-61.
94. Botden, S.M.B.I., et al., Augmented versus virtual reality laparoscopic simulation: What is the difference? A comparison of the ProMIS augmented reality laparoscopic simulator versus LapSim virtual reality laparoscopic simulator. World Journal of Surgery, 2007. 31(4): p. 764-772.
95. Córdova-Domínguez, J.A., et al., Pilot study of a virtual simulation model to teach the lumbar sympathetic block procedure. Revista Mexicana de Anestesiologia, 2007. 30(2): p. 61-66.
96. Cosman, P.H., et al., Skills acquired on virtual reality laparoscopic simulators transfer into the operating room in a blinded, randomised, controlled trial. Studies in health technology and informatics, 2007. 125: p. 76-81.
97. Neequaye, S.K., et al., Identification of skills common to renal and iliac endovascular procedures performed on a virtual reality simulator. Eur J Vasc Endovasc Surg, 2007. 33(5): p. 525-32.
98. Park, J., et al., Randomized controlled trial of virtual reality simulator training: transfer to live patients. Am J Surg, 2007. 194(2): p. 205-11.
99. Roche, P.L., et al., Multi-school collaboration to develop and test nutrition computer modules for pediatric residents. J Am Diet Assoc, 2007. 107(9): p. 1586-9.
100. Ryan, G., et al., Online CME: an effective alternative to face-to-face delivery. Med Teach, 2007. 29(8): p. e251-7.
101. Taylor, D.R. and P. Maniar, The Children's Advocacy Project of Philadelphia's Cap4Kids survey: an innovative tool for pediatrician--community-based organization collaboration. Clinical Pediatrics, 2007. 46(6): p. 512-517.
102. Towbin, A.J., B. Paterson, and P.J. Chang, A Computer-Based Radiology Simulator as a Learning Tool to Help Prepare First-Year Residents for Being On Call. Academic Radiology, 2007. 14(10): p. 1271-1283.
103. von Sternberg, N., et al., Learning by doing virtually. Int J Oral Maxillofac Surg, 2007. 36(5): p. 386-90.
104. Xiao, Y., et al., Video-based training increases sterile-technique compliance during central venous catheter insertion. Crit Care Med, 2007. 35(5): p. 1302-6.
105. Akl, E.A., et al., An educational game for teaching clinical practice guidelines to Internal Medicine residents: development, feasibility and acceptability. BMC Med Educ, 2008. 8: p. 50.
106. Bell, D.S., et al., Knowledge retention after an online tutorial: A randomized educational experiment among resident physicians. Journal of General Internal Medicine, 2008. 23(8): p. 1164-1171.
107. Beyea, J.A., et al., Evaluation of a particle repositioning maneuver Web-based teaching module. Laryngoscope, 2008. 118(1): p. 175-80.
108. Binstadt, E., et al., Simulator Training Improves Fiber-optic Intubation Proficiency among Emergency Medicine Residents. Academic Emergency Medicine, 2008. 15(11): p. 1211-1214.
109. Botden, S.M., et al., The importance of haptic feedback in laparoscopic suturing training and the additive value of virtual reality simulation. Surg Endosc, 2008. 22(5): p. 1214-22.
110. Braeckman, L.A., A.M. Fieuw, and H.J. Van Bogaert, A web- and case-based learning program for postgraduate students in occupational medicine. Int J Occup Environ Health, 2008. 14(1): p. 51-6.

111. Bryce, E., et al., E-learning of infection control: it's contagious. Can J Infect Control, 2008. 23(4): p. 228, 230, 232 passim.
112. Casebeer, L., et al., A controlled trial of the effectiveness of internet continuing medical education. BMC Med, 2008. 6: p. 37.
113. Chenkin, J., et al., Procedures can be learned on the Web: a randomized study of ultrasound-guided vascular access training. Academic Emergency Medicine, 2008. 15(10): p. 949-954.
114. Cook, D.A., et al., Introducing resident doctors to complexity in ambulatory medicine. Med Educ, 2008. 42(8): p. 838-48.
115. Cook, D.A., et al., Adapting web-based instruction to residents' knowledge improves learning efficiency: a randomized controlled trial. J Gen Intern Med, 2008. 23(7): p. 985-90.
116. Coughlan, J. and S.S. Morar, Development of a tool for evaluating multimedia for surgical education. J Surg Res, 2008. 149(1): p. 94-100.
117. Criley, J.M., et al., Innovative web-based multimedia curriculum improves cardiac examination competency of residents. J Hosp Med, 2008. 3(2): p. 124-33.
118. Dy, S.M., et al., Evaluation of a web-based palliative care pain management module for housestaff. J Pain Symptom Manage, 2008. 36(6): p. 596-603.
119. El Saadawi, G.M., et al., A natural language intelligent tutoring system for training pathologists: implementation and evaluation. Adv Health Sci Educ Theory Pract, 2008. 13(5): p. 709-22.
120. Farrar, M., et al., Teaching doctors how to diagnose paroxysmal events: a comparison of two educational methods. Medical Education, 2008. 42(9): p. 909-914 6p.
121. Harting, B., et al., Computer-based simulation as a teaching tool for residents treating patients with cancer-related pain crises. Quality Management in Health Care, 2008. 17(3): p. 192-199.
122. Hugenholtz, N.I., et al., Effectiveness of e-learning in continuing medical education for occupational physicians. Occup Med (Lond), 2008. 58(5): p. 370-2.
123. Klass, D., et al., Training on a vascular interventional simulator: an observational study. Eur Radiol, 2008. 18(12): p. 2874-8.
124. Kulier, R., et al., Harmonising evidence-based medicine teaching: a study of the outcomes of e-learning in five European countries. BMC Med Educ, 2008. 8: p. 27.
125. Lendvay, T.S., et al., VR robotic surgery: randomized blinded study of the dV-Trainer robotic simulator. Stud Health Technol Inform, 2008. 132: p. 242-4.
126. Maddaus, M.A., et al., Rotation as a Course: Lessons Learned from Developing a Hybrid Online/On-Ground Approach to General Surgical Resident Education. Journal of Surgical Education, 2008. 65(2): p. 112-116.
127. McFadden, P. and A. Crim, Comparison of the Effectiveness of Interactive Didactic Lecture Versus Online Simulation-Based CME Programs Directed at Improving the Diagnostic Capabilities of Primary Care Practitioners. Journal of Continuing Education in the Health Professions, 2008. 36(1): p. 32-37.
128. Rubio, E.I., et al., Effect of an audience response system on resident learning and retention of lecture material. AJR Am J Roentgenol, 2008. 190(6): p. W319-22.
129. Saxena, A., et al., Merging old school methods with new technology to improve skills in cardiac auscultation. Hospital Physician, 2008. 44(6): p. 43-48.
130. Sears, K.E., J.E. Cohen, and J. Drope, Comprehensive evaluation of an online tobacco control continuing education course in Canada. J Contin Educ Health Prof, 2008. 28(4): p. 235-40.
131. Shirai, Y., et al., Prospective randomized study on the use of a computer-based endoscopic simulator for training in esophagogastroduodenoscopy. Journal of Gastroenterology and Hepatology (Australia), 2008. 23(7): p. 1046-1050.
132. Sim, J. and A. Radloff, Enhancing reflective practice through online learning: Impact on clinical practice. Biomedical Imaging and Intervention Journal, 2008. 4(1).
133. Soyinka, A.S., et al., Enhancing laparoscopic performance with the LTS3E: a computerized hybrid physical reality simulator. Fertil Steril, 2008. 90(5): p. 1988-94.

134. Verdaasdonk, E.G., et al., Transfer validity of laparoscopic knot-tying training on a VR simulator to a realistic environment: a randomized controlled trial. Surg Endosc, 2008. 22(7): p. 1636-42.
135. Weeks, D.L. and D.M. Molsberry, Pediatric advanced life support re-training by videoconferencing compared to face-to-face instruction: a planned non-inferiority trial. Resuscitation, 2008. 79(1): p. 109-17.
136. Blumberg, D.M., H.A. Quigley, and H.R. Goldberg, Quantitative and qualitative evaluation of a web-based, interactive approach for teaching the management of angle-closure glaucoma. J Glaucoma, 2009. 18(2): p. 107-13.
137. Cook, C.B., et al., Development of computer-based training to enhance resident physician management of inpatient diabetes. J Diabetes Sci Technol, 2009. 3(6): p. 1377-87.
138. Cook, D.A., et al., Measuring motivational characteristics of courses: applying Keller's instructional materials motivation survey to a web-based course. Acad Med, 2009. 84(11): p. 1505-9.
139. Copenhaver, B.R., et al., Gradient echo MRI: implementation of a training tutorial for intracranial hemorrhage diagnosis. Neurology, 2009. 72(18): p. 1576-81.
140. Danila, R., et al., Objective evaluation of minimally invasive surgical skills for transplantation. Surgeons using a virtual reality simulator. Chirurgia (Bucur), 2009. 104(2): p. 181-5.
141. Feudner, E.M., et al., Virtual reality training improves wet-lab performance of capsulorhexis: results of a randomized, controlled study. Graefes Arch Clin Exp Ophthalmol, 2009. 247(7): p. 955-63.
142. Haycock, A.V., et al., Colonoscopy skills transfer from a second-generation virtual reality simulator to patients: A multinational randomised blinded controlled trial. Gut, 2009. 58: p. A50.
143. Jensen, M.L., et al., Using e-learning for maintenance of ALS competence. Resuscitation, 2009. 80(8): p. 903-908.
144. Jones, P.E. and K.E. Mulitalo, A novel case-based approach to continuing medical education using a virtual patient record. Journal of Physician Assistant Education (Physician Assistant Education Association), 2009. 20(3): p. 28-30.
145. Jones, R.B., et al., Use of live interactive webcasting for an international postgraduate module in ehealth: case study evaluation. J Med Internet Res, 2009. 11(4): p. e46.
146. Kash, K.M., et al., ACGME competencies in neurology: web-based objective simulated computerized clinical encounters. Neurology, 2009. 72(10): p. 893-8.
147. Kerfoot, B.P., Y. Fu, and E.M. Genega, Interactive spaced education versus web-based modules for teaching histopathology diagnostic skills to urology residents: A randomized controlled trial. Laboratory Investigation, 2009. 89: p. 114A.
148. Kleinert, H.L., et al., Improving residents' understanding of issues, comfort levels, and patient needs regarding screening for and diagnosing Down syndrome. American Journal of Obstetrics & Gynecology, 2009. 201(3): p. 328.e1-6.
149. Koch, L.H., et al., Randomized comparison of virtual microscopy and traditional glass microscopy in diagnostic accuracy among dermatology and pathology residents. Hum Pathol, 2009. 40(5): p. 662-7.
150. Kruglikova, I., et al., The impact of constructive feedback in gastrointestinal-endoscopy training: Assessment of learning curves using a high-fidelity virtual reality simulator in controlled randomized study. Scandinavian Journal of Gastroenterology, 2009. 44: p. 24.
151. Kulier, R., et al., The effectiveness of a clinically integrated e-learning course in evidence-based medicine: a cluster randomised controlled trial. BMC Med Educ, 2009. 9(1): p. 21.
152. Larsen, C.R., et al., Effect of virtual reality training on laparoscopic surgery: randomised controlled trial. BMJ, 2009. 338: p. b1802.
153. Lerner, C., et al., Enhancing pediatric safety: assessing and improving resident competency in life-threatening events with a computer-based interactive resuscitation tool. Pediatr Radiol, 2009. 39(7): p. 703-9.
154. Moldovanu, R., et al., Training on a virtual reality simulator--is it really possible a correct evaluation of the surgeons' experience? Rev Med Chir Soc Med Nat Iasi, 2009. 113(3): p. 780-7.

155. Mulgrew, B., et al., An evaluation of the effects of a web-based modular teaching programme, housed within a virtual learning environment on orthodontic training for specialist registrars. Journal of orthodontics, 2009. 36(3): p. 167-176.
156. Sarker, S.K., et al., A decision-making learning and assessment tool in laparoscopic cholecystectomy. Surg Endosc, 2009. 23(1): p. 197-203.
157. Schlickum, M.K., et al., Systematic video game training in surgical novices improves performance in virtual reality endoscopic surgical simulators: a prospective randomized study. World J Surg, 2009. 33(11): p. 2360-7.
158. Schroter, S., et al., Evaluation of an online Diabetes Needs Assessment Tool (DNAT) for health professionals: a randomised controlled trial. Trials, 2009. 10: p. 63.
159. Sendag, F., et al., Virtual reality technology in laparoscopic surgical education. Gynecological Surgery, 2009. 6: p. S83.
160. Welke, T.M., et al., Personalized oral debriefing versus standardized multimedia instruction after patient crisis simulation. Anesth Analg, 2009. 109(1): p. 183-9.
161. Woo, M.Y., et al., Effectiveness of a novel training program for emergency medicine residents in ultrasound-guided insertion of central venous catheters. CJEM, 2009. 11(4): p. 343-8.
162. Yamagami, T., et al., Development of a portable training tool for simulating visceral angiographic procedures for beginners. Cardiovasc Intervent Radiol, 2009. 32(3): p. 412-6.
163. Bittner, J.G.t., et al., Face and construct validity of a computer-based virtual reality simulator for ERCP. Gastrointest Endosc, 2010. 71(2): p. 357-64.
164. Burch, J. and M.D. Wiles, Evaluation of an online bronchoscopy simulator as an educational tool. Anaesthesia, 2010. 65(8): p. 867.
165. Ferlitsch, A., et al., Effect of virtual endoscopy simulator training on performance of upper gastrointestinal endoscopy in patients: a randomized controlled trial. Endoscopy, 2010. 42(12): p. 1049-56.
166. Hardy, A.B., L. Jones, and J. Kastelik, Development of a web-based training programme for respiratory physicians in Yorkshire. Clin Med (Lond), 2010. 10(4): p. 344-8.
167. Hauge, L.S., et al., Web-based curriculum improves residents' knowledge of health care business. J Am Coll Surg, 2010. 211(6): p. 777-83.
168. Haycock, A., et al., Web-based learning in gastroenterology: Reallife learning outcomes from a training and assessment module in lesion recognition at capsule endoscopy. Gut, 2010. 59: p. A9.
169. Henderson, B.A., et al., Evaluation of the virtual mentor cataract training program. Ophthalmology, 2010. 117(2): p. 253-8.
170. Jacobs, K., J. Ivy, and P. Judson, Advancement of surgical skills with implementation of virtual reality simulation training. Gynecologic Oncology, 2010. 116(3): p. S144.
171. Kahol, K., et al., Quantitative evaluation of retention of surgical skills learned in simulation. J Surg Educ, 2010. 67(6): p. 421-6.
172. Perkins, G.D., et al., The effect of pre-course e-learning prior to advanced life support training: a randomised controlled trial. Resuscitation, 2010. 81(7): p. 877-81.
173. Sitzmann, T., et al., The effects of technical difficulties on learning and attrition during online training. J Exp Psychol Appl, 2010. 16(3): p. 281-92.
174. Stasek, J.E., et al., Use of an internet-based instruction module in teaching complex clinical subject matter to physicians-in-training. American Journal of Respiratory and Critical Care Medicine, 2010. 181(1).
175. Sullivan, M.D., et al., Randomized Trial of Web-based Training About Opioid Therapy for Chronic Pain. Clinical Journal of Pain, 2010. 26(6): p. 512-517.
176. Talib, N., et al., Effective educational instruction in preventive oral health: hands-on training versus web-based training. Pediatrics, 2010. 125(3): p. 547-53.
177. Westmorel, G.R., et al., Web-based training in geriatrics for medical residents: a randomized controlled trial using standardized patients to assess outcomes. Journal of the American Geriatrics Society, 2010. 58(6): p. 1163-1169.

178. Wheeler, B., et al., Interactive computer-based simulator for training in blade navigation and targeting in myringotomy. Comput Methods Programs Biomed, 2010. 98(2): p. 130-9.
179. Wong, R., et al., Evaluation of a web-based, self-learning module on the 'resident as a manager' for junior residents. Medical Education, Supplement, 2010. 44: p. 31-32.
180. Zitto, T., et al., Continuous medical education: Teaching infectology using the internet. International Journal of Infectious Diseases, 2010. 14: p. e432-e433.
181. Andersen, C., T.N. Winding, and M.S. Vesterby, Development of simulated arthroscopic skills: A randomized trial of virtual-reality training of 21 orthopedic surgeons. Acta Orthopaedica, 2011. 82(1): p. 90-95.
182. Bensalem-Owen, M., et al., Education Research: Evaluating the use of podcasting for residents during EEG instruction: A pilot study. Neurology, 2011. 77(8): p. e42-4.
183. Branzetti, J.B., et al., A novel online didactic curriculum helps improve knowledge acquisition among non-emergency medicine rotating residents. Acad Emerg Med, 2011. 18(1): p. 53-9.
184. Crochet, P., et al., Deliberate practice on a virtual reality laparoscopic simulator enhances the quality of surgical technical skills. Ann Surg, 2011. 253(6): p. 1216-22.
185. Davids, M.R., U.M. Chikte, and M.L. Halperin, Development and evaluation of a multimedia e-learning resource for electrolyte and acid-base disorders. Adv Physiol Educ, 2011. 35(3): p. 295-306.
186. Fuller, C.D., et al., Prospective evaluation of an online atlas-based educational intervention on head and neck organ-at-risk (OAR) and Lymph node level (LNL) contouring: A pilot feasibility study using web-based feedback and analytic software. International Journal of Radiation Oncology Biology Physics, 2011. 81(2): p. S161.
187. Gordon, M., et al., Is a short E-learning course effective at improving paediatric prescribing skills amongst UK foundation doctors? An open label randomised controlled trial. Medical Education, Supplement, 2011. 45: p. 14-15.
188. Gordon, M., M. Chandratilake, and P. Baker, Improved junior paediatric prescribing skills after a short e-learning intervention: a randomised controlled trial. Arch Dis Child, 2011. 96(12): p. 1191-4.
189. Gorrindo, T., et al., Web-based simulation in psychiatry residency training: a pilot study. Acad Psychiatry, 2011. 35(4): p. 232-237.
190. Iwata, N., et al., Construct validity of the LapVR virtual-reality surgical simulator. Surg Endosc, 2011. 25(2): p. 423-8.
191. Jerath, A., et al., An interactive online 3D model of the heart assists in learning standard transesophageal echocardiography views. Can J Anaesth, 2011. 58(1): p. 14-21.
192. Kobak, K.A., et al., Web-based training in early autism screening: results from a pilot study. Telemed J E Health, 2011. 17(8): p. 640-4.
193. Llambi, L., et al., Teaching tobacco cessation skills to Uruguayan physicians using information and communication technologies. J Contin Educ Health Prof, 2011. 31(1): p. 43-8.
194. McEvoy, M.M., et al., Virtual patients: an effective educational intervention to improve paediatric basic specialist trainee education in the management of suspected child abuse? Ir Med J, 2011. 104(8): p. 250-2.
195. Mooney, E., et al., Comparative diagnostic accuracy in virtual dermatopathology. Skin Res Technol, 2011. 17(2): p. 251-5.
196. O'Connor, M.E., E.W. Brown, and L.O. Lewin, An Internet-based education program improves breastfeeding knowledge of maternal-child healthcare providers. Breastfeed Med, 2011. 6(6): p. 421-7.
197. Platz, E., et al., Are live instructors replaceable? Computer vs. classroom lectures for EFAST training. J Emerg Med, 2011. 40(5): p. 534-8.
198. Pourm, A., R. Lucas, and M. Nouraie, A comparison of asynchronous web-based learning and traditional educational conferences on medical knowledge acquisition among emergency medicine residents. Academic Emergency Medicine, 2011. 18(5): p. S96-S97.
199. Ramanathan, R., et al., Mixed methods evaluation of an international internet-based continuing medical education course for pediatric HIV providers in Pune, India. Educ Health (Abingdon), 2011. 24(1): p. 540.

200. Rank, M.A., G.W. Volcheck, and T. Swagger, A randomized controlled trial of a web-based educational intervention for teaching allergen immunotherapy. Journal of Allergy and Clinical Immunology, 2011. 127(2): p. AB42.
201. Schopf, T. and V. Flytkjaer, Doctors and nurses benefit from interprofessional online education in dermatology. BMC Med Educ, 2011. 11: p. 84.
202. Shaw, T., et al., Impact on clinical behavior of face-to-face continuing medical education blended with online spaced education: a randomized controlled trial. J Contin Educ Health Prof, 2011. 31(2): p. 103-8.
203. Spice, R., et al., Design and implementation of an online course on research methods in palliative care: lessons learned. J Palliat Med, 2011. 14(4): p. 413-9.
204. Wolpin, S., et al., Evaluation of online training on the prevention of venous thromboembolism. Vasc Endovascular Surg, 2011. 45(2): p. 146-56.
205. Wu, R., et al., Evaluation of a web-based interactive heart failure patient simulation: a pilot study. Can J Cardiol, 2011. 27(3): p. 369-75.
206. Zendejas, B., et al., Simulation-based mastery learning improves patient outcomes in laparoscopic inguinal hernia repair: a randomized controlled trial. Ann Surg, 2011. 254(3): p. 502-9; discussion 509-11.
207. Adams, B.J., F. Margaron, and B.J. Kaplan, Comparing video games and laparoscopic simulators in the development of laparoscopic skills in surgical residents. J Surg Educ, 2012. 69(6): p. 714-7.
208. Alfieri, J., et al., Development and impact evaluation of an e-learning radiation oncology module. Int J Radiat Oncol Biol Phys, 2012. 82(3): p. e573-80.
209. Austin, E.W., et al., The relationships of information efficacy and media literacy skills to knowledge and self-efficacy for health-related decision making. J Am Coll Health, 2012. 60(8): p. 548-54.
210. Bateman, H.E., et al., Innovative virtual interactive teaching tool "Arthur" for clinical diagnosis of musculoskeletal diseases. J Clin Rheumatol, 2012. 18(3): p. 151-2.
211. Bittner, J., et al., Reality vs. virtual reality-a comparison of colonoscopy simulators. Surgical Endoscopy and Other Interventional Techniques, 2012. 26: p. S292.
212. Burden, C., et al., Validation of virtual reality simulation for obstetric ultrasonography: a prospective cross-sectional study. Simul Healthc, 2012. 7(5): p. 269-73.
213. Furlong, M., et al., Surgical pathology resident 'bootcamp': Innovative web-based modules and preparatory simulation for the surgical pathology rotation. American Journal of Clinical Pathology, 2012. 138: p. A110.
214. Giglioli, S., et al., Self-directed deliberate practice with virtual fiberoptic intubation improves initial skills for anesthesia residents. Minerva Anestesiol, 2012. 78(4): p. 456-61.
215. Havsteen, I., et al., E-learn computed tomographic angiography: a proposed educational tool for computed tomographic angiography in acute stroke. J Stroke Cerebrovasc Dis, 2012. 21(8): p. 684-8.
216. Holmboe, E.S., et al., Comparative trial of a web-based tool to improve the quality of care provided to older adults in residency clinics: modest success and a tough road ahead. Acad Med, 2012. 87(5): p. 627-34.
217. Innes, A., F. Kelly, and L. McCabe, An evaluation of an online postgraduate dementia studies program. Gerontol Geriatr Educ, 2012. 33(4): p. 364-82.
218. Kerfoot, B.P. and H. Baker, An online spaced-education game for global continuing medical education: a randomized trial. Ann Surg, 2012. 256(1): p. 33-8.
219. Korndorffer, J.R., Jr., et al., Effective home laparoscopic simulation training: a preliminary evaluation of an improved training paradigm. Am J Surg, 2012. 203(1): p. 1-7.
220. Kulier, R., et al., Effectiveness of a clinically integrated e-learning course in evidence-based medicine for reproductive health training: a randomized trial. JAMA, 2012. 308(21): p. 2218-25.
221. Larsen, C.R., et al., The efficacy of virtual reality simulation training in laparoscopy: a systematic review of randomized trials. Acta Obstet Gynecol Scand, 2012. 91(9): p. 1015-28.

222. Lee, M.O., et al., A medical simulation-based educational intervention for emergency medicine residents in neonatal resuscitation. Acad Emerg Med, 2012. 19(5): p. 577-85.
223. Lonn, L., et al., Virtual reality simulation training in a high-fidelity procedure suite: operator appraisal. J Vasc Interv Radiol, 2012. 23(10): p. 1361-6 e2.
224. McIntosh, K.S., N.V. Khanna, and J.C. Gregor, Computer based virtual reality colonoscopy simulation improves patient based colonoscopy performance. Gastrointestinal Endoscopy, 2012. 75(4): p. AB364.
225. Niazi, A.U., et al., Ultrasound-guided regional anesthesia performance in the early learning period: effect of simulation training. Reg Anesth Pain Med, 2012. 37(1): p. 51-4.
226. Palan, J., et al., The use of a virtual learning environment in promoting virtual journal clubs and case-based discussions in trauma and orthopaedic postgraduate medical education: The Leicester experience. Journal of Bone & Joint Surgery, British Volume, 2012. 94(9): p. 1170-1175.
227. Platts, D.G., et al., The use of computerised simulators for training of transthoracic and transoesophageal echocardiography. The future of echocardiographic training? Heart Lung Circ, 2012. 21(5): p. 267-74.
228. Rinewalt, D., H. Du, and J.M. Velasco, Evaluation of a novel laparoscopic simulation laboratory curriculum. Surgery, 2012. 152(4): p. 550-4; discussion 554-6.
229. Samakar, K., et al., Evaluating virtual reality simulator training on surgical residents perceptions of stress: A randomized controlled trial. Surgical Endoscopy and Other Interventional Techniques, 2012. 26: p. S287.
230. Satterwhite, T., et al., Microsurgery education in residency training: validating an online curriculum. Ann Plast Surg, 2012. 68(4): p. 410-4.
231. Shah, M.N., et al., A novel internet-based geriatric education program for emergency medical services providers. J Am Geriatr Soc, 2012. 60(9): p. 1749-54.
232. Simonian, S.M., et al., Asynchronous learning: A comparison of knowledge acquisition between traditional conference lectures versus iTunesU Mp4 distance learning among emergency medicine residents. Annals of Emergency Medicine, 2012. 60(5): p. S168.
233. Stolbach, A., et al., Successful short and long-term educational outcomes in residents using internet toxidromes curriculum. Clinical Toxicology, 2012. 50(7): p. 627-628.
234. Tamler, R., et al., Durability of the effect of online diabetes training for medical residents on knowledge, confidence, and inpatient glycemia. J Diabetes, 2012. 4(3): p. 281-90.
235. Thompson, J.S., et al., Knowledge of quality performance measures associated with endoscopy among gastroenterology trainees and the impact of a web-based intervention. Gastrointestinal Endoscopy, 2012. 76(1): p. 100-106.e4.
236. Vaidya, A., et al., Improving the management of diabetes in hospitalized patients: the results of a computer-based house staff training program. Diabetes Technol Ther, 2012. 14(7): p. 610-8.
237. Walsh, C.M., et al., Virtual reality simulation training for health professions trainees in gastrointestinal endoscopy. Cochrane Database Syst Rev, 2012(6): p. CD008237.
238. Wiet, G.J., et al., Virtual temporal bone dissection system: OSU virtual temporal bone system: development and testing. Laryngoscope, 2012. 122 Suppl 1: p. S1-12.
239. Woodworth, G., E. Chen, and J.L. Horn, Effectiveness of computer-based video and simulation in ultrasound-guided regional anesthesia training. Regional Anesthesia and Pain Medicine, 2012. 37(6).
240. Zendejas, B., et al., Mastery learning simulation-based curriculum for laparoscopic TEP inguinal hernia repair. J Surg Educ, 2012. 69(2): p. 208-14.
241. Ahlborg, L., et al., Simulator training and non-technical factors improve laparoscopic performance among OBGYN trainees. Acta Obstet Gynecol Scand, 2013. 92(10): p. 1194-201.
242. Audcent, T.A., et al., Development and evaluation of global child health educational modules. Pediatrics, 2013. 132(6): p. e1570-6.
243. Bernstein, H.H., et al., Evaluation of a national Bright Futures oral health curriculum for pediatric residents. Acad Pediatr, 2013. 13(2): p. 133-9.

244. Bharathan, R., et al., Psychomotor skills and cognitive load training on a virtual reality laparoscopic simulator for tubal surgery is effective. Eur J Obstet Gynecol Reprod Biol, 2013. 169(2): p. 347-52.
245. Bordman, R., et al., Curriculum to enhance pharmacotherapeutic knowledge in family medicine: interprofessional coteaching and web-based learning. Can Fam Physician, 2013. 59(11): p. e493-8.
246. Brick, K.E., et al., Comparison of virtual microscopy and glass slide microscopy among dermatology residents during a simulated in-training examination. Journal of cutaneous pathology, 2013. 40(9): p. 807-811.
247. Damp, J., et al., Effects of transesophageal echocardiography simulator training on learning and performance in cardiovascular medicine fellows. J Am Soc Echocardiogr, 2013. 26(12): p. 1450-1456 e2.
248. Feist, M., et al., Methods and effects of a case-based pediatric gastroenterology online curriculum. J Pediatr Gastroenterol Nutr, 2013. 56(2): p. 161-5.
249. Guiahi, M., et al., Evaluation of an e-learning family planning curriculum for obstetrics and gynecology residents. Contraception, 2013. 88(2): p. 310-311.
250. Gurusamy, K.S., et al., Virtual reality training for surgical trainees in laparoscopic surgery. Cochrane Database of Systematic Reviews, 2013(8): p. N.PAG-N.PAG.
251. Harris, J.M., Jr. and H. Sun, A randomized trial of two e-learning strategies for teaching substance abuse management skills to physicians. Acad Med, 2013. 88(9): p. 1357-62.
252. Hashimoto, D.A., et al., A randomized controlled trial to assess if deliberate practice on a virtual reality simulator can achieve ''expert'' surgical performance. Surgical Endoscopy and Other Interventional Techniques, 2013. 27: p. S338.
253. Hearty, T., et al., Orthopaedic resident preparedness for closed reduction and pinning of pediatric supracondylar fractures is improved by e-learning: a multisite randomized controlled study. Journal of Bone & Joint Surgery, American Volume, 2013. 95(17): p. e1261-7.
254. Kerr, B., et al., Feasibility of scenario-based simulation training versus traditional workshops in continuing medical education: a randomized controlled trial. Med Educ Online, 2013. 18: p. 21312.
255. Kobak, K.A., et al., Web-based therapist training on cognitive behavior therapy for anxiety disorders: a pilot study. Psychotherapy (Chic), 2013. 50(2): p. 235-47.
256. Leblanc, J., et al., A comparison of orthopaedic resident performance on surgical fixation of an ulnar fracture using virtual reality and synthetic models. Journal of Bone & Joint Surgery, American Volume, 2013. 95(9): p. e601-6.
257. Lendvay, T.S., et al., Virtual reality robotic surgery warm-up improves task performance in a dry laboratory environment: a prospective randomized controlled study. J Am Coll Surg, 2013. 216(6): p. 1181-92.
258. Patel, S.G., et al., Learning curves using cumulative sum analysis (CUSUM) for the histologic characterization of diminutive colorectal polyps using a computer-based teaching module and narrow band imaging (NBI) videos: Implications for resect and discard strategy. Gastrointestinal Endoscopy, 2013. 77(5): p. AB145.
259. Patel, V., et al., Implementation of an interactive virtual-world simulation for structured surgeon assessment of clinical scenarios. J Am Coll Surg, 2013. 217(2): p. 270-9.
260. Rapoport, M.J., et al., Online continuing medical education in geriatric psychiatry: Preliminary evaluation of a national canadian program. American Journal of Geriatric Psychiatry, 2013. 21(3): p. S161-S162.
261. Robinson, W.P., et al., Simulation-based training to teach open abdominal aortic aneurysm repair to surgical residents requires dedicated faculty instruction. J Vasc Surg, 2013. 58(1): p. 247-53 e1-2.
262. Saleh, G.M., et al., The development of a virtual reality training programme for ophthalmology: repeatability and reproducibility (part of the International Forum for Ophthalmic Simulation Studies). Eye (Lond), 2013. 27(11): p. 1269-74.

263. Schirmer, C.M., et al., Virtual reality-based simulation training for ventriculostomy: an evidence-based approach. Neurosurgery, 2013. 73 Suppl 1: p. 66-73.
264. Shenoi, R., et al., Interactive spaced online education in pediatric trauma. Academic Emergency Medicine, 2013. 20(5): p. S81.
265. Sperl-Hillen, J., et al., Virtual diabetes education improves resident physician knowledge and performance: A cluster-randomized trial. Diabetes, 2013. 62: p. A172-A173.
266. Williams, J., T.S. Sato, and B. Policeni, Pulmonary embolism teaching file: a simple pilot study for rapidly increasing pulmonary embolism recognition among new residents using interactive cross-sectional imaging. Acad Radiol, 2013. 20(8): p. 1048-51.
267. Yudkowsky, R., et al., Practice on an augmented reality/haptic simulator and library of virtual brains improves residents' ability to perform a ventriculostomy. Simul Healthc, 2013. 8(1): p. 25-31.
268. Zendejas, B., et al., Comparative effectiveness of virtual reality versus box trainer simulators for laparoscopic surgery training: A systematic review & meta-analysis. Journal of Surgical Research, 2013. 179(2).
269. Zhang, Y., et al., Training for percutaneous renal access on a virtual reality simulator. Chin Med J (Engl), 2013. 126(8): p. 1528-31.
270. Zhu, H., et al., Virtual reality simulator for training urologists on transurethral prostatectomy. Chin Med J (Engl), 2013. 126(7): p. 1220-3.
271. Akdemir, A., F. Sendag, and M.K. Oztekin, Laparoscopic virtual reality simulator and box trainer in gynecology. Int J Gynaecol Obstet, 2014. 125(2): p. 181-5.
272. Allen, G.B., et al., A multitiered strategy of simulation training, kit consolidation, and electronic documentation is associated with a reduction in central line–associated bloodstream infections. American Journal of Infection Control, 2014. 42(6): p. 643-648.
273. Azer, N., et al., 'iBIM' - Internet-based interactive modules: an easy and interesting learning tool for general surgery residents. Canadian Journal of Surgery, 2014. 57(2): p. E31-5.
274. Brown, M. and A. Bullock, Evaluating PLATO: postgraduate teaching and learning online. Clin Teach, 2014. 11(1): p. 10-4.
275. Cannon, W.D., et al., Improving residency training in arthroscopic knee surgery with use of a virtual-reality simulator. A randomized blinded study. Journal of Bone & Joint Surgery, American Volume, 2014. 96(21): p. 1798-1806.
276. Connolly, A.M., et al., 'Beyond Milestones': a randomised controlled trial evaluating an innovative digital resource teaching quality observation of normal child development. J Paediatr Child Health, 2014. 50(5): p. 393-8.
277. Cook, D.A., W.G. Thompson, and K.G. Thomas, Test-enhanced web-based learning: optimizing the number of questions (a randomized crossover trial). Acad Med, 2014. 89(1): p. 169-75.
278. Davids, M.R., U.M. Chikte, and M.L. Halperin, Effect of improving the usability of an e-learning resource: a randomized trial. Adv Physiol Educ, 2014. 38(2): p. 155-60.
279. Franchi, C., et al., E-learning to improve the drug prescribing in the hospitalized elderly patients: the ELICADHE feasibility pilot study. Aging Clin Exp Res, 2014. 26(4): p. 435-43.
280. Gorrindo, T., et al., Autonomic arousal and learning in Web-based simulation: a feasibility study. J Contin Educ Health Prof, 2014. 34 Suppl 1: p. S17-22.
281. Huang, K.J., et al., Application of international videoconferences for continuing medical education programs related to laparoscopic surgery. Telemed J E Health, 2014. 20(2): p. 157-60.
282. Jensen, K., et al., Simulation-based training for thoracoscopic lobectomy: a randomized controlled trial: virtual-reality versus black-box simulation. Surg Endosc, 2014. 28(6): p. 1821-9.
283. Kang, S.G., et al., The Tube 3 module designed for practicing vesicourethral anastomosis in a virtual reality robotic simulator: determination of face, content, and construct validity. Urology, 2014. 84(2): p. 345-50.
284. Kawamura, H., et al., A study on comparison of learning effects between a board game and a lecture about infection control. Yakugaku Zasshi, 2014. 134(7): p. 839-849.

285. Khan, M.W., et al., Laparoscopic skills maintenance: a randomized trial of virtual reality and box trainer simulators. J Surg Educ, 2014. 71(1): p. 79-84.
286. Kiely, D.J., et al., A randomized controlled trial of a proficiency-based, virtual-reality robotic simulation curriculum to teach robotic suturing. Gynecologic Oncology, 2014. 133: p. 193.
287. Krupinski, E., et al., Understanding Visual Search Patterns of Dermatologists Assessing Pigmented Skin Lesions Before and After Online Training. Journal of Digital Imaging, 2014. 27(6): p. 779-785.
288. Kunin, M., K.N. Julliard, and T.E. Rodriguez, Comparing face-to-face, synchronous, and asynchronous learning: postgraduate dental resident preferences. J Dent Educ, 2014. 78(6): p. 856-66.
289. Levine, M., B. Sellers, and C. Pearce, A novel electronic asynchronous webbased learning resource increases trauma knowledge and is favorable to emergency medicine residents. Academic Emergency Medicine, 2014. 21(5): p. S71.
290. Lichtman, A.S., et al., A validation study: Does use of an interactive computer based laparoscopic hysterectomy trainer expand cognitive surgical knowledge of ob/gyn trainees beyond that of traditional educational tools? Journal of Minimally Invasive Gynecology, 2014. 21(6): p. S87.
291. Marlow, N., et al., Laparoscopic skills acquisition: a study of simulation and traditional training. ANZ J Surg, 2014. 84(12): p. 976-80.
292. Matyal, R., et al., Simulator-based transesophageal echocardiographic training with motion analysis: a curriculum-based approach. Anesthesiology, 2014. 121(2): p. 389-99.
293. Meuser, T.M., et al., The instructional impact of the American Medical Association's Older Drivers Project online curriculum. Gerontol Geriatr Educ, 2014. 35(1): p. 64-85.
294. Moon, M.R., et al., Web-based education on primary care of the adolescent patient: Comparison of internal medicine and pediatric programs use and test scores. Journal of Adolescent Health, 2014. 54(2): p. S53.
295. Nevin, C.R., et al., Gamification as a tool for enhancing graduate medical education. Postgrad Med J, 2014. 90(1070): p. 685-93.
296. Palter, V.N. and T.P. Grantcharov, Individualized deliberate practice on a virtual reality simulator improves technical performance of surgical novices in the operating room: a randomized controlled trial. Ann Surg, 2014. 259(3): p. 443-8.
297. Ruparel, R.K., et al., Assessment of virtual reality robotic simulation performance by urology resident trainees. J Surg Educ, 2014. 71(3): p. 302-8.
298. Samuelson, S.T., Simulation as a set-up for technical mastery: Can a high-fidelity virtual warm-up improve resident performance of fiberoptic intubation? Anesthesia and Analgesia, 2014. 118(5): p. S13.
299. Satterwhite, T., et al., The Stanford Microsurgery and Resident Training (SMaRT) Scale: validation of an on-line global rating scale for technical assessment. Ann Plast Surg, 2014. 72 Suppl 1: p. S84-8.
300. Schreuder, H.W., et al., Validation of a novel virtual reality simulator for robotic surgery. ScientificWorldJournal, 2014. 2014: p. 507076.
301. Seagull, F.J. and D.M. Rooney, Filling a void: developing a standard subjective assessment tool for surgical simulation through focused review of current practices. Surgery, 2014. 156(3): p. 718-22.
302. Sperl-Hillen, J., et al., Educating resident physicians using virtual case-based simulation improves diabetes management: a randomized controlled trial. Academic medicine : journal of the Association of American Medical Colleges, 2014. 89(12): p. 1664-1673.
303. Stanley, K., K. Dillard, and R. Naylor, Improving diabetes education for pediatric residents with an online module. Journal of Investigative Medicine, 2014. 62(4): p. 711-712.
304. Szwajcer, A., K. Macdonald, and B. Kvern, Health Literacy Training for Family Medicine Residents. Journal of the Canadian Health Libraries Association (JCHLA), 2014. 35(3): p. 128-132.
305. Thepwongsa, I., et al., Online continuing medical education (CME) for GPs: does it work? A systematic review. Australian Family Physician, 2014. 43(10): p. 717-721.

306. Vogell, A., V. Wright, and K. Wright, An evaluation of the utility of robotic virtual reality simulation in gynecologic resident surgical education. Journal of Minimally Invasive Gynecology, 2014. 21(6): p. S84-S85.
307. White, I., et al., A virtual reality endoscopic simulator augments general surgery resident cancer education as measured by performance improvement. Journal of Cancer Education, 2014. 29(2): p. 333-336.
308. York, S.L., et al., Development and evaluation of cesarean section surgical training using computer-enhanced visual learning. Med Teach, 2014. 36(11): p. 958-64.
309. Akhtar, K., et al., Training safer orthopedic surgeons. Construct validation of a virtual-reality simulator for hip fracture surgery. Acta Orthop, 2015. 86(5): p. 616-21.
310. Alwaal, A., et al., Transfer of skills on LapSim virtual reality laparoscopic simulator into the operating room in urology. Urology Annals, 2015. 7(2): p. 172-176.
311. Amori, R.E., et al., Implementation of computer based learning modules in diabetes for first year internal medicine residents: A review of the first year of use. Endocrine Reviews, 2015. 36.
312. Beaulieu, Y., et al., Bedside ultrasound training using web-based e-learning and simulation early in the curriculum of residents. Critical Ultrasound Journal, 2015. 7(1).
313. Boespflug, A., et al., Enhancement of Customary Dermoscopy Education With Spaced Education e-Learning: A Prospective Controlled Trial. JAMA Dermatol, 2015. 151(8): p. 847-53.
314. Bonevski, B., et al., An internet based approach to improve general practitioners' knowledge and practices: the development and pilot testing of the "ABC's of vitamin D" program. Int J Med Inform, 2015. 84(6): p. 413-22.
315. Brateanu, A., et al., Impact of an online spaced repetition-learning module on residents' medical knowledge competency. Journal of General Internal Medicine, 2015. 30: p. S498-S499.
316. Close, A., et al., Beta Test of Web-Based Virtual Patient Decision-Making Exercises for Residents Demonstrates Discriminant Validity and Learning. J Surg Educ, 2015. 72(6): p. e130-6.
317. DeBonis, K., et al., Viability of a Web-Based Module for Teaching Electrocardiogram Reading Skills to Psychiatry Residents: Learning Outcomes and Trainee Interest. Acad Psychiatry, 2015. 39(6): p. 645-8.
318. Deraniyagala, R., et al., Usability study of the EduMod eLearning Program for contouring nodal stations of the head and neck. Pract Radiat Oncol, 2015. 5(3): p. 169-75.
319. Diehhl, L.A., et al., Effectiveness of a serious game for medical education on insulin therapy for diabetes: Randomized controlled trial. Diabetology and Metabolic Syndrome, 2015. 7: p. 71.
320. Diehl, L.A., et al., User Assessment of "InsuOnLine," a Game to Fight Clinical Inertia in Diabetes: A Pilot Study. Games Health J, 2015. 4(5): p. 335-43.
321. Dolan, B.M., M.A. Yialamas, and G.T. McMahon, A Randomized Educational Intervention Trial to Determine the Effect of Online Education on the Quality of Resident-Delivered Care. J Grad Med Educ, 2015. 7(3): p. 376-81.
322. Garcia de Diego, L., M. Cuervo, and J.A. Martinez, Development of a learning-oriented computer assisted instruction designed to improve skills in the clinical assessment of the nutritional status: a pilot evaluation. PLoS One, 2015. 10(5): p. e0126345.
323. Giudice, E.L., et al., Online Versus In-Person Screening, Brief Intervention, and Referral to Treatment Training in Pediatrics Residents. J Grad Med Educ, 2015. 7(1): p. 53-8.
324. Hansen, C., et al., E-learning education for ultrasound-guided peripheral blocks. A prospective study evaluating individual increment in learning curves during a one-month trial period. Regional Anesthesia and Pain Medicine, 2015. 40(5): p. e110.
325. Huang, C., et al., Face and content validity of a virtual-reality simulator for myringotomy with tube placement. J Otolaryngol Head Neck Surg, 2015. 44(1): p. 40.
326. Jagroep, S.R., An evaluation of physicians for reproductive health's adolescent reproductive and sexual health (ARSH) E-learning course with feedback from learners and residency coordinators. Journal of Adolescent Health, 2015. 56(2): p. S113-S114.

327. Kelly, D.M., et al., A Structured Educational Curriculum Including Online Training Positively Impacts American Board of Surgery In-Training Examination Scores. J Surg Educ, 2015. 72(5): p. 811-7.
328. Khan, T., et al., Comparison of classroom didactic teaching (DT) and computer based self-learning (Sl) in recognizing narrow band imaging (NBI) patterns for diminutive polyp (DP) histology characterization. Gastrointestinal Endoscopy, 2015. 81(5): p. AB325.
329. Kiely, D.J., et al., Virtual reality robotic surgery simulation curriculum to teach robotic suturing: a randomized controlled trial. J Robot Surg, 2015. 9(3): p. 179-86.
330. Kuo, F.-R., et al., Web-Based Learning System for Developing and Assessing Clinical Diagnostic Skills for Dermatology Residency Program. Educational Technology & Society, 2015. 19(3): p. 194-206.
331. Liu, D.B., et al., Teaching of the Society for Fetal Urology grading system for pediatric hydronephrosis is improved by e-Learning using Computer Enhanced Visual Learning (CEVL): A multi-institutional trial. Journal of Pediatric Urology, 2015. 11(4): p. 184.e1-184.e8.
332. McGrath, J., et al., Virtual alternative to the oral examination for emergency medicine residents. West J Emerg Med, 2015. 16(2): p. 336-43.
333. Mount, H.R., et al., Text messaging to improve resident knowledge: a randomized controlled trial. Fam Med, 2015. 47(1): p. 37-42.
334. Nesterowicz, K., S.M. Fereshtehnejad, and S. Edelbring, e-learning in continuing pharmacy education is effective and just as accepted as on-site learning. Pharmacy Education, 2015. 15(1): p. 22-26.
335. Salvado, J.A., et al., Validation of a high-fidelity model in ureteroscopy incorporating hand motion analysis. Int Urol Nephrol, 2015. 47(8): p. 1265-9.
336. Smelt, J., et al., Simulation-based learning of transesophageal echocardiography in cardiothoracic surgical trainees: A prospective, randomized study. J Thorac Cardiovasc Surg, 2015. 150(1): p. 22-5.
337. Tomaz, J.B., et al., Effectiveness of an online Problem-Based learning curriculum for training family medical doctors in Brazil. Educ Health (Abingdon), 2015. 28(3): p. 187-93.
338. Van Es, S.L., et al., Cytopathology whole slide images and adaptive tutorials for postgraduate pathology trainees: a randomized crossover trial. Hum Pathol, 2015. 46(9): p. 1297-305.
339. Van Laar, E.S., et al., Improving hematologist/oncologist knowledge and confidence in managing hemophilia through online educational interventions. Blood, 2015. 126(23): p. 5593.
340. Whittle, A.E., et al., Addressing Adolescent Substance Use: Teaching Screening, Brief Intervention, and Referral to Treatment (SBIRT) and Motivational Interviewing (MI) to Residents. Substance Abuse, 2015. 36(3): p. 325-331.
341. Wilkening, G.L., et al., Evaluation of virtual patient technology for interdisciplinary education of psychiatry residents. Journal of Pharmacy Practice, 2015. 28(3): p. 348-349.
342. Wingo, M.T., et al., Enhancing motivation with the "virtual" supervisory role: a randomized trial. BMC Med Educ, 2015. 15: p. 76.
343. Andersen, S.A., et al., The effect of self-directed virtual reality simulation on dissection training performance in mastoidectomy. Laryngoscope, 2016. 126(8): p. 1883-8.
344. Boody, B., et al., Validation of a Web-Based Curriculum for Resident Education in Orthopedic Surgery. J Surg Educ, 2016. 73(6): p. 1060-1065.
345. Bowe, S.N., et al., Programmatic Assessment of a Comprehensive Quality Improvement Curriculum in an Otolaryngology Residency. Otolaryngol Head Neck Surg, 2016. 155(5): p. 729-732.
346. Carbonne, B. and I. Sabri-Kaci, Assessment of an e-learning training program for cardiotocography analysis: a multicentre randomized study. Eur J Obstet Gynecol Reprod Biol, 2016. 197: p. 111-5.
347. Casey, D.B., D. Stewart, and M.I. Vidovich, Diagnostic coronary angiography: initial results of a simulation program. Cardiovasc Revasc Med, 2016. 17(2): p. 102-5.
348. Del Cura-Gonzalez, I., et al., Effectiveness of a strategy that uses educational games to implement clinical practice guidelines among Spanish residents of family and community medicine (e-EDUCAGUIA project): a clinical trial by clusters. Implement Sci, 2016. 11: p. 71.

349. DelSignore, L.A., et al., Test-Enhanced E-Learning Strategies in Postgraduate Medical Education: A Randomized Cohort Study. J Med Internet Res, 2016. 18(11): p. e299.
350. Dennis, E., et al., Validation of an electronic program for pathologist training in the interpretation of a complex companion diagnostic immunohistochemical assay. Hum Pathol, 2016. 56: p. 194-203.
351. Diena, C., et al., Development of an online asynchronous sleep medicine educational program. Sleep, 2016. 39: p. A393.
352. Edrich, T., et al., A Comparison of Web-Based with Traditional Classroom-Based Training of Lung Ultrasound for the Exclusion of Pneumothorax. Anesth Analg, 2016. 123(1): p. 123-8.
353. Elledge, R., et al., Use of a virtual learning environment for training in maxillofacial emergencies: impact on the knowledge and attitudes of staff in accident and emergency departments. Br J Oral Maxillofac Surg, 2016. 54(2): p. 166-9.
354. Farokhi, M.R., et al., E-learning or in-person approaches in continuous medical education: A comparative study. IIOAB Journal, 2016. 7: p. 472-476.
355. Fulton, N., et al., Simulation-Based Training May Improve Resident Skill in Ultrasound-Guided Biopsy. AJR Am J Roentgenol, 2016. 207(6): p. 1329-1333.
356. Garcia-Rodriguez, J.A. and T. Donnon, Using Comprehensive Video-Module Instruction as an Alternative Approach for Teaching IUD Insertion. Fam Med, 2016. 48(1): p. 15-20.
357. Golding, E., et al., Effect of a communication skills intervention on resident comfort and knowledge of discussing goals of care and advance care planning. Journal of Clinical Oncology, 2016. 34(29): p. 50-50.
358. Jensen, U.J., et al., Virtual reality training in coronary angiography and its transfer effect to real-life catheterisation lab. EuroIntervention, 2016. 11(13): p. 1503-10.
359. Kalu, N., et al., Impact of a multicomponent screening, brief intervention, and referral to treatment (SBIRT) training curriculum on a medical residency program. Subst Abus, 2016. 37(1): p. 242-7.
360. Kilgore, B., et al., Improving Resident Knowledge of Spacers. Clin Pediatr (Phila), 2016. 55(11): p. 1050-3.
361. Kornegay, J.G., et al., Development and implementation of an asynchronous emergency medicine residency curriculum using a web-based platform. Intern Emerg Med, 2016. 11(8): p. 1115-1120.
362. Larkin, A., et al., Effectiveness of online medical education on clinical decision-making in hypoparathyroidism management. Endocrine Reviews, 2016. 37(2).
363. Law, M., et al., Evaluation of a National Online Educational Program in Geriatric Psychiatry. Acad Psychiatry, 2016. 40(6): p. 923-927.
364. Le Marne, F.A., et al., Evaluation of an E-learning resource on approach to the first unprovoked seizure. J Paediatr Child Health, 2016. 52(9): p. 896-900.
365. Locketz, G., et al., Anatomy specific virtual simulation in temporal bone dissection. Otolaryngology - Head and Neck Surgery (United States), 2016. 155: p. P47.
366. Lubarda, J., P. Chatterjee, and T. Vlahovic, Can online medical education improve management of challenging cases of onychomycosis? Journal of the American Academy of Dermatology, 2016. 74(5): p. AB133.
367. Luetsch, K. and J. Burrows, Certainty rating in pre-and post-tests of study modules in an online clinical pharmacy course - A pilot study to evaluate teaching and learning. BMC Med Educ, 2016. 16(1): p. 267.
368. Magin, P.J., et al., Reducing general practice trainees' antibiotic prescribing for respiratory tract infections: an evaluation of a combined face-to-face workshop and online educational intervention. Educ Prim Care, 2016. 27(2): p. 98-105.
369. Marston, B.A., et al., The virtual rheumatology clinic: Virtual patients for resident education in rheumatology. Arthritis and Rheumatology, 2016. 68: p. 1466-1467.

370. Martin, K.D., et al., Comparison of Three Virtual Reality Arthroscopic Simulators as Part of an Orthopedic Residency Educational Curriculum. Iowa Orthop J, 2016. 36: p. 20-5.
371. Prasad, M., et al., A novel, mobile, electronic curriculum based on adult learning theory is noninferior to a traditional didactic lecture-based curriculum. European Heart Journal, 2016. 37: p. 1265.
372. Samuelson, S.T., et al., Simulation as a set-up for technical proficiency: Can a virtual warm-up improve live fibre-optic intubation? British Journal of Anaesthesia, 2016. 116(3): p. 398-404.
373. Serr, M., et al., [CME MMC: Evaluation of a continuous medical education tool by e-learning from the morbi-mortality conferences of the Burgundy]. Journal de Gynecologie Obstetrique et Biologie de la Reproduction, 2016.
374. Taveira-Gomes, T., et al., What Are We Looking for in Computer-Based Learning Interventions in Medical Education? A Systematic Review. Journal of medical Internet research, 2016. 18(8): p. e204.
375. Te Pas, E., et al., Blended learning in CME: the perception of GP trainers. Educ Prim Care, 2016. 27(3): p. 217-24.
376. Thomsen, A.S.S.S., et al., Investigating inter-procedural transfer of surgical skills using virtual-reality simulation. Investigative Ophthalmology and Visual Science, 2016. 57(12): p. 5827.
377. Van Dam, M.J. and E.W.M.T. Ter Braak, Development and evaluation of an e-learning to enhance advanced care planning discussion by residents in a university medical center, a pilot study. Intensive Care Medicine Experimental, 2016. 4.
378. Verhovsek, M.M., et al., Pilot study of online learning modules for hemoglobinopathy education in canadian hematology training programs. Blood, 2016. 128(22).
379. Waterman, B.R., et al., Simulation Training Improves Surgical Proficiency and Safety During Diagnostic Shoulder Arthroscopy Performed by Residents. Orthopedics, 2016. 39(3): p. e479-85.
380. Zaveri, P.P., et al., Virtual Reality for Pediatric Sedation: A Randomized Controlled Trial Using Simulation. Cureus, 2016. 8(2): p. e486.
381. Andersen, S.A., et al., Mapping the plateau of novices in virtual reality simulation training of mastoidectomy. Laryngoscope, 2017. 127(4): p. 907-914.
382. Barthelemy, F.X., et al., ECG interpretation in Emergency Department residents: An update and e-learning as a resource to improve skills. European Journal of Emergency Medicine, 2017. 24(2): p. 149-156.
383. Bassil, A., et al., Operative and diagnostic hysteroscopy: A novel learning model combining new animal models and virtual reality simulation. Eur J Obstet Gynecol Reprod Biol, 2017. 211: p. 42-47.
384. Breimer, G.E., et al., Simulation-based education for endoscopic third ventriculostomy: A comparison between virtual and physical training models. Operative Neurosurgery, 2017. 13(1): p. 89-95.
385. Cullinan, S., D. O'Mahony, and S. Byrne, Use of an e-Learning Educational Module to Better Equip Doctors to Prescribe for Older Patients: A Randomised Controlled Trial. Drugs Aging, 2017. 34(5): p. 367-374.
386. Diehl, L.A., et al., InsuOnline, an Electronic Game for Medical Education on Insulin Therapy: A Randomized Controlled Trial With Primary Care Physicians. J Med Internet Res, 2017. 19(3): p. e72.
387. Ens, A., K. Janzen, and M.R. Palmert, Development of an Online Learning Module to Improve Pediatric Residents' Confidence and Knowledge of the Pubertal Examination. J Adolesc Health, 2017. 60(3): p. 292-298.
388. Gillespie, E.F., et al., Multi-institutional Randomized Trial Testing the Utility of an Interactive Three-dimensional Contouring Atlas Among Radiation Oncology Residents. Int J Radiat Oncol Biol Phys, 2017. 98(3): p. 547-554.
389. Hughes, S., et al., Improvement in knowledge of diagnostic criteria of narcolepsy among neurologists following participation in an online medical education activity. Sleep, 2017. 40: p. A248-A249.
390. Ivy, A.S., et al., Improving the Identification of Neonatal Encephalopathy: Utility of a Web-Based Video Tool. Am J Perinatol, 2017. 34(5): p. 520-522.

391. Jacobs, Z.G., et al., An e-learning module on chronic low back pain in older adults: Effect on medical resident attitudes, confidence, knowledge, and practice patterns. Journal of General Internal Medicine, 2017. 32(2): p. S112.
392. Kaban, L.B., et al., Evaluation of Oral and Maxillofacial Surgery Residents' Operative Skills: Feasibility and Engagement Study Using SIMPL Software for a Mobile Phone. Journal of Oral & Maxillofacial Surgery (02782391), 2017. 75(10): p. 2041-2047.
393. Khan, T., et al., Didactic training vs. computer-based self-learning in the prediction of diminutive colon polyp histology by trainees: a randomized controlled study. Endoscopy, 2017. 49(12): p. 1243-1250.
394. Krishnamachari, B., et al., Video Education on Hereditary Breast and Ovarian Cancer (HBOC) for Physicians: an Interventional Study. J Cancer Educ, 2017. 32(2): p. N.PAG-N.PAG.
395. Kutzin, J.M., Z. Milligan, and S. Chawla, Using Simulation to Conduct a Usability Study of Wearable Technology. Clinical Simulation in Nursing, 2017. 13(2): p. 64-70.
396. Laborde, C.J., et al., Evaluation of a novel tablet application for improvement in colonoscopy training and mentoring (with video). Gastrointest Endosc, 2017. 85(3): p. 559-565 e1.
397. Larkin, A., et al., Effect of an online continuing medical education and clinician coaching quality improvement initiative on antiplatelet medication adherence and hospital readmissions in patients with acute coronary syndrome. Journal of the American College of Cardiology, 2017. 69(11): p. 2489.
398. Le Pape, P., et al., E-learning to improve paediatric parenteral nutrition knowledge? a pilot study in two hospitals. European Journal of Hospital Pharmacy, 2017. 24: p. A124.
399. Lee, G.I. and M.R. Lee, Can a virtual reality surgical simulation training provide a self-driven and mentor-free skills learning? Investigation of the practical influence of the performance metrics from the virtual reality robotic surgery simulator on the skill learning and associated cognitive workloads. Surgical Endoscopy and Other Interventional Techniques, 2017: p. 1-11.
400. Locketz, G.D., et al., Anatomy-Specific Virtual Reality Simulation in Temporal Bone Dissection: Perceived Utility and Impact on Surgeon Confidence. Otolaryngol Head Neck Surg, 2017. 156(6): p. 1142-1149.
401. Madani, A., et al., Measuring intra-operative decision-making during laparoscopic cholecystectomy: validity evidence for a novel interactive Web-based assessment tool. Surg Endosc, 2017. 31(3): p. 1203-1212.
402. Matos, J., et al., Spaced education in medical residents: An electronic intervention to improve competency and retention of medical knowledge. PLoS One, 2017. 12(7): p. e0181418.
403. Middleton, R.M., et al., Simulation-Based Training Platforms for Arthroscopy: A Randomized Comparison of Virtual Reality Learning to Benchtop Learning. Arthroscopy, 2017. 33(5): p. 996-1003.
404. Mistraletti, G., et al., Neurological assessment with validated tools in general ICU: multicenter, randomized, before and after, pragmatic study to evaluate the effectiveness of an e-learning platform for continuous medical education. Minerva Anestesiol, 2017. 83(2): p. 145-154.
405. Mitchell, M.A., et al., Education for the mind and the heart? Changing residents' attitudes about addressing unhealthy alcohol use. Substance Abuse, 2017. 38(1): p. 40-42.
406. Newcomb, L.K., et al., Correlation of Virtual Reality Simulation and Dry Lab Robotic Technical Skills. J Minim Invasive Gynecol, 2017. 23(5): p. S27.
407. Paquette, J., et al., Virtual Laparoscopy Simulation: a Promising Pedagogic Tool in Gynecology. JSLS, 2017. 21(3).
408. Patel, R. and R. Dennick, Simulation based teaching in interventional radiology training: is it effective? Clin Radiol, 2017. 72(3): p. 266 e7-266 e14.
409. Patel, S.N., et al., Assessment of a Tele-education System to Enhance Retinopathy of Prematurity Training by International Ophthalmologists-in-Training in Mexico. Ophthalmology, 2017. 124(7): p. 953-961.
410. Paul, C.L., et al., Poor uptake of an online intervention in a cluster randomised controlled trial of online diabetes education for rural general practitioners. Trials, 2017. 18(1): p. 137.

411. Real, F.J., et al., A Virtual Reality Curriculum for Pediatric Residents Decreases Rates of Influenza Vaccine Refusal. Acad Pediatr, 2017. 17(4): p. 431-435.
412. Robinson, T., et al., New Approaches to Continuing Medical Education: a QStream (spaced education) Program for Research Translation in Ovarian Cancer. J Cancer Educ, 2017. 32(3): p. 476-482.
413. Rotimi, O., et al., Remote Teaching of Histopathology Using Scanned Slides via Skype Between the United Kingdom and Nigeria. Archives of Pathology & Laboratory Medicine, 2017. 141(2): p. 298-300.
414. Schmidt, M.W., et al., Learning from the surgeon's real perspective – First-person view versus laparoscopic view in e-learning for training of surgical skills? Study protocol for a randomized controlled trial. International Journal of Surgery Protocols, 2017. 3: p. 7-13.
415. Thomsen, A.S., et al., Operating Room Performance Improves after Proficiency-Based Virtual Reality Cataract Surgery Training. Ophthalmology, 2017. 124(4): p. 524-531.
416. Wilkening, G.L., et al., Evaluation of Branched-Narrative Virtual Patients for Interprofessional Education of Psychiatry Residents. Acad Psychiatry, 2017. 41(1): p. 71-75.
417. Williams, K., N.H. Abd-Hamid, and Y. Perkhounkova, Transitioning Communication Education to an Interactive Online Module Format. J Contin Educ Nurs, 2017. 48(7): p. 320-328.
418. Wong, A., et al., Impact of online toxicology training on health professionals: the Global Educational Toxicology Uniting Project (GETUP). Clin Toxicol (Phila), 2017. 55(9): p. 981-985.
